# Monitoring activities of receptor tyrosine kinases using a universal adapter in genetically encoded split TEV assays

**DOI:** 10.1007/s00018-018-03003-2

**Published:** 2019-01-08

**Authors:** Jan P. Wintgens, Sven P. Wichert, Luksa Popovic, Moritz J. Rossner, Michael C. Wehr

**Affiliations:** 1 Department of Psychiatry and Psychotherapy, University Hospital, LMU Munich, Nussbaumstr. 7, 80336 Munich, Germany; 2 Systasy Bioscience GmbH, Adams-Lehmann-Str. 56, 80797 Munich, Germany

**Keywords:** Cell-based assay, Receptor tyrosine kinases, TEV protease, Split TEV recruitment assay, Lapatinib

## Abstract

Receptor tyrosine kinases (RTKs) play key roles in various aspects of cell biology, including cell-to-cell communication, proliferation and differentiation, survival, and tissue homeostasis, and have been implicated in various diseases including cancer and neurodevelopmental disorders. Ligand-activated RTKs recruit adapter proteins through a phosphotyrosine (p-Tyr) motif that is present on the RTK and a p-Tyr-binding domain, like the Src homology 2 (SH2) domain found in adapter proteins. Notably, numerous combinations of RTK/adapter combinations exist, making it challenging to compare receptor activities in standardised assays. In cell-based assays, a regulated adapter recruitment can be investigated using genetically encoded protein–protein interaction detection methods, such as the split TEV biosensor assay. Here, we applied the split TEV technique to robustly monitor the dynamic recruitment of both naturally occurring full-length adapters and artificial adapters, which are formed of clustered SH2 domains. The applicability of this approach was tested for RTKs from various subfamilies including the epidermal growth factor (ERBB) family, the insulin receptor (INSR) family, and the hepatocyte growth factor receptor (HGFR) family. Best signal-to-noise ratios of ligand-activated RTK receptor activation was obtained when clustered SH2 domains derived from GRB2 were used as adapters. The sensitivity and robustness of the RTK recruitment assays were validated in dose-dependent inhibition assays using the ERBB family-selective antagonists lapatinib and WZ4002. The RTK split TEV recruitment assays also qualify for high-throughput screening approaches, suggesting that the artificial adapter may be used as universal adapter in cell-based profiling assays within pharmacological intervention studies.

**Electronic supplementary material:**

The online version of this article (10.1007/s00018-018-03003-2) contains supplementary material, which is available to authorized users.

## Introduction

Receptor tyrosine kinases (RTKs) are type I transmembrane protein receptors and respond, with few exceptions, to extracellular cues. RTK-mediated signalling regulates key processes of cell biology, including intercellular communication, proliferation and differentiation, cell survival and metabolism, cell migration, and cell cycle control [1, 2]. Further, RTK signalling is also implicated in central nervous system (CNS) and peripheral nervous system (PNS) development [3]. RTKs share a similar architecture, which consists of ligand-binding domains in the extracellular region, a single alpha-helix crossing the membrane and, in the cytoplasm, a juxtamembrane domain, a protein tyrosine kinase domain, and a carboxyl (C-) terminal regulatory region [2]. To date, 58 receptor tyrosine kinases in humans have been described, which can be divided into 20 subfamilies [2]. Together with the G protein-coupled receptors (GPCR), RTKs are the most important receptor class in human cells and represent the most relevant drug targets in the cell [4]. However, only 3% of marketed drugs target kinases as such, suggesting that the development of cell-based assays with broader applicability and robustness may contribute to better medicines.

Upon ligand stimulation, RTKs dimerise that causes a kinase domain-mediated phosphorylation of the cytoplasmic receptor tails in *trans*, followed by phosphorylation-dependent recruitment of adapter proteins. The ligand-induced binding between an RTK and an adapter is commonly mediated by phosphotyrosine (p-Tyr) motifs, which are present in the cytoplasmic tail of an RTK and serve as docking sites for adapter proteins containing phospho-binding modules, such as the Src homology 2 (SH2) domain or phosphotyrosine-binding (PTB) domain [5]. The SH2 domain is the largest class of p-Tyr recognition domains and comprises 120 different domains in 110 proteins [6]. The specificity of binding between a given SH2 domain and a p-Tyr docking site is mediated by the SH2 domain itself and the sequence of the p-Tyr motif, which is defined by the p-Tyr residue and its flanking residues [7].

One of the best studied RTK families is the Erb-b2 receptor tyrosine kinases (ERBB) family comprising the epidermal growth factor (EGFR, also known as HER1 in humans) and ERBB2, ERBB3, and ERBB4 (also known as HER2, HER3, and HER4 in humans). The ERBB family has been linked to the development of, amongst other tissues, skin, heart, CNS, and PNS, and is widely implicated in human diseases, such as cancer and neurodevelopmental disorders including schizophrenia [2, 3]. Upon ligand stimulation, ERBB receptors homo- or heterodimerise, depending on the cellular context. ERBB2, however, is the preferred dimerisation partner for the other ERBB receptors and does not respond to ligands [8, 9]. ERBB3 needs to heterodimerise to initiate downstream signalling, as the kinase domain lacks catalytic activity, and its preferred partner for heterodimerisation is ERBB2 [10]. The major ligand for EGFR is the epidermal growth factor (EGF), whereas ERBB3 and ERBB4 are predominantly activated by neuregulins (NRG1-4) [11]. NRG binding to the receptor is mediated by the EGF-like domain (EGFld), which on its own can stimulate ERBB3 and ERBB4 receptors [11].

The activity of ERBB receptors can be measured using genetically encoded bioassays, such as split TEV [12, 13]. This technique allows assessing dynamic protein–protein interactions in living cells and is based on the functional complementation of the tobacco etch virus (TEV) protease coupled to genetically encoded reporters, like the GAL4/UAS system combined with firefly luciferase (Fluc) as reporter gene readout. The assay system was, for example, applied to monitor phosphorylation-dependent interactions of ERBB4 with the adapters PIK3R1 (regulatory subunit of PI3K), SHC1, and GRB2, as well as the EGFld-induced homodimer formation of ERBB4 and the heterodimer formation of ERBB2 and ERBB3 [13–16].

In this work, we describe the application of split TEV-based RTK recruitment assays that provide a universal adapter recruitment strategy for robust and flexible cell-based assays applicable to dose–response profiling and high-throughput screening (HTS) assays. To do this, disease-relevant RTKs such as the complete ERBB family, the insulin growth factor 1 receptor (IGF1R), and mesenchymal epithelial transition proto-oncogene (MET, also known as c-MET or hepatocyte growth factor receptor) were selected and tested in split TEV dose–response assays for their assay performance, both using endogenous full-length adapters and artificial adapters consisting of clustered SH2 domains. The latter ones were designed to increase flexibility, robustness, and eventually lower the number of adapters needed for assaying various RTK activities, thus improving the comparability among the receptors tested. Notably, the artificial p-Tyr sensor based on the SH2 domain clustering derived from GRB2 displayed an improved signal-to-noise ratio in RTK recruitment assays. Furthermore, the p-Tyr sensor has been validated in dose–response assays using the ERBB family antagonists lapatinib and WZ4002, as well as the IGF1R inhibitor linsitinib and the MET antagonist foretinib. As expected, lapatinib and WZ4002 inhibited ERBB family assays. However, challenging IGF1R and MET split TEV recruitment assays with lapatinib did not have an effect, demonstrating the sensitivity of our approach. Taken together, we established robust split TEV recruitment assays to sensitively monitor RTK receptor activities in living cells using a universal adapter protein as recruitment sensor.

## Materials and methods

### Plasmids

ORFs were PCR-amplified using the Pwo proofreading DNA Polymerase (Roche) and BP-recombined into the pDONR/Zeo plasmid using Gateway recombination cloning (Life Technologies). Each entry vector was control digested using BsrGI, which releases the insert, and finally verified by sequencing. LR recombination that was used to shuffle the ORFs from the entry vectors into the split TEV destination vectors (either pcDNA_attR1-ORF-attR2-NTEV-tcs-GV-2xHA_DEST or pcDNA3_attR1-ORF-attR2-CTEV-2xHA_DEST). The generation of the human ORFs for EGFR, ERBB2, ERBB3, ERBB4, SHC1, GRB2, and PIK3R1 has been described previously [16]. IGF1R and MET were obtained as pENTR plasmids from Harvard Plasmid ID (clone HsCD00040705) and Addgene (as part of the CCSB-Broad Human Kinase ORF Collection [17]), respectively. Concatenated ORFs of clustered SH2 domains that are flanked by attL1 and attL2 sites were synthesised by GenScript, USA. These sequences were provided in a pUC57 vector backbone harbouring a kanamycin resistance gene, allowing LR recombination cloning with destination vectors carrying an ampicillin resistance gene. DNA and protein sequences of clustered SH2 domains are provided in Fig. S1.

### Cell culture

PC12 Tet-Off cells (Clontech, 631134, termed PC12 cells for simplicity) were maintained in DMEM medium (1 g/l glucose, Lonza) supplemented with 10% FCS, 5% horse serum (HS, Thermo Fisher Scientific), and 100 U/ml each of penicillin and streptomycin and 2 mM GlutaMAX. To starve PC12 cells, 2% FCS, 100 U/ml each of penicillin and streptomycin and 2 mM GlutaMAX, but no HS, were added to the DMEM medium (1 g/l glucose). PC12 cells were grown on poly-l-lysine (Sigma) coated surfaces for maintenance and experiments. A549 cells (ATCC, CCL-185) were cultured in DMEM medium (4.5 g/l glucose) supplemented with 10% FCS and 100 U/ml each of penicillin and streptomycin and 2 mM GlutaMAX. T-47D cells (ATCC, HTB-133) were cultured in RPMI 1640 medium supplemented with human insulin (f.c. 125 µg/l) (Sigma-Aldrich), 10% FCS, and 100 U/ml each of penicillin and streptomycin and 2 mM GlutaMAX. Cells were cultured at 37 °C and 5% CO_2_.

### Biochemistry

For assessing the phosphorylation levels of EGFR, A549 cells were starved overnight using 1% FCS, pre-incubated with increasing concentration of compounds [i.e. lapatinib (Selleckchem) or WZ4002 (Sigma-Aldrich)] at semi-logarithmic scale for 1 h, and stimulated with 30 ng/ml EGF (Sigma-Aldrich) for 5 min. Likewise, T-47D were starved overnight using 0.5% FCS, pre-incubated with increasing concentration of compounds (i.e. lapatinib or WZ4002) at semi-logarithmic scale for 1 h, and stimulated with 10 ng/ml EGF-like domain (Sigma-Aldrich) for 5 min. Split TEV expression plasmids were transfected into PC12 cells using Lipofectamine 2000 (Thermo Fisher Scientific) according to the manufacturer’s instructions. Cells were washed 1 × with PBS and lysed in a Triton-X lysis buffer (1% Triton-X100, 50 mM Tris pH7.5, 150 mM NaCl, 1 mM EGTA) containing the Complete Protease Inhibitor Cocktail (Roche) and PhosSTOP phosphatase inhibitor (Roche). Briefly, cells were lysed and kept on ice for 10 min, sonicated 3 × for 10 s at 4 °C, and denatured for 10 min at 70 °C. The Mini-PROTEAN Tetra Electrophoresis System and Trans-Blot Turbo Blotting System (both Bio-Rad) were used for running and blotting protein gels. Chemiluminescence detection of proteins by Western blot analysis was performed using the Western LightningPlus-ECL kit (PerkinElmer). HA-tagged proteins were visualised using an HA antibody (clone 3F10, dilution 1:250, No. 11 867 423 001, Roche). The ERBB2-V5 fusion was stained using a V5 antibody (clone D3H8Q, dilution 1:1000, Cell Signaling Technology). Phosphorylation levels of EGFR and ERBB4 were assayed using p-EGFR-Y1068 (clone D7A5, dilution 1:500, No. 3777, Cell Signaling Technology) and p-ERBB4-Y1284 antibodies (clone 21A9, dilution 1:500, No. 4757, Cell Signaling Technology). Total EGFR and ERBB4 protein levels were determined using an anti-EGFR antibody (clone A-10, dilution 1:1000, sc-373746, Santa Cruz Biotechnology) and an anti-ERBB4 antibody (clone E200, dilution 1:1000, ab32375, Abcam). Tubulin levels were determined using an anti-tubulin antibody (dilution 1:2000, No. T 5168, Sigma-Aldrich). For quantification, phosphorylation levels of p-EGFR relative to EGFR as well as p-ERBB4 relative to ERBB4 were calculated using the Lukemiller protocol (http://lukemiller.org/index.php/2010/11/analyzing-gels-and-western-blots-with-image-j/). Assays were run in triplicate.

### Immunocytochemistry

On day 1, 1 Mio PC12 cells were plated on coverslips coated with poly-l-lysine (PLL) and placed in a six-well plate. On day 2, cells were transfected with EGFR-Glink-NTEV-tevS-GV-2HA, ERBB2-var1-V5, ERBB3-Glink-NTEV-tevS-GV-2HA, or ERBB4-JMa-CVT1-Glink-NTEV-tevS-GV-2HA using Lipofectamine 2000. On day 3, 50 µl of 1 × TBS was gently added per coverslip and removed twice to wash the cells, followed by fixation in 4% PFA diluted in 1 × TBS for 10 min, washed again once in 1 × TBS and then permeabilised in TBS/0.1% Triton X-100 for 5 min. Subsequently, cells were washed three times with 1 × TBS, then blocked in blocking buffer (3% BSA, 0.1% Triton X-100 in 1 × TBS) for 30 min at room temperature, and again washed three times with 1 × TBS. The HA antibody (Poly9023, BioLegend) and the V5 antibody (clone D3H8Q, Cell Signaling Technology), respectively, were diluted in blocking buffer (1:1000) and added to the cells and incubated overnight at 4 °C. After another three washing steps using 1 × TBS, the secondary antibody (Alexa 594 anti-rabbit, 1:500, Abcam, ab150160) diluted in blocking buffer was added to the cells and incubated for 1 h at room temperature. Coverslips were washed three times in 1 × TBS, dipped into ddH_2_O to remove traces of salt, mounted on microscope slides, and sealed with ProLong Gold Antifade Mountant with Dapi (ThermoFisher Scientific, P36935). Slides were imaged on a Zeiss Observer Z.1 microscope.

### Split TEV recruitment end-point assays

Split TEV recruitment assays were run in six replicates per condition in 96-well plates. 50,000 PC12 cells per well were seeded onto poly-l-lysine (PLL)-coated plates. The next day, cells were transfected with assay plasmids using Lipofectamine 2000. For transfection-based assays, no antibiotics were added to the medium. Per 96-well, a receptor-NTEV-tcs-GV fusion plasmid (10 ng), an adapter-CTEV fusion plasmid for either SHC1 (10 ng), SH2(SHC1) (10 ng), SH2(GRB2) (10 ng), SH2(mix) (10 ng), PIK3R1(50 ng), SH2(PIK3R1) (50 ng), or GRB2 (2.5 ng), and an Fluc reporter plasmid (10 ng, Fluc driven by 10x clustered upstream activating sequences coupled to a minimal CMV promoter, 10 × UAS-minCMVp) were used. Additionally, 1 ng of a plasmid constitutively expressing an EYFP that is fused to nuclear localisation sequence (EYFPnuc) driven by a CMV promoter was transfected per 96-well to assess transfection efficiencies (EYFPnuc). In detail, assay plasmids were diluted in 30 µl Opti-MEM (Thermo Fisher Scientific), vortexed, and mixed with 0.2 µl Lipofectamine 2000 per well. The DNA/Lipofectamine/Opti-MEM mix was incubated for 20 min. The medium was removed from the wells and the DNA/Lipofectamine/Opti-MEM mix was added onto the cells. After 2 h of incubation, the maintenance medium without antibiotics was added to the Opti-MEM mix. On day 2, the maintenance medium was replaced by starvation medium. After 16–20 h (day 3), six wells per condition were stimulated with EGF (30 ng/ml, Sigma-Aldrich), EGFld (10 ng/ml, Sigma-Aldrich), IGF1 (100 ng/ml, PeproTech), and HGF (100 ng/ml, Sigma-Aldrich), while six wells were left non-stimulated. 16 h later (day 4), the medium was removed, and cells were lysed using Passive Lysis Buffer (Promega) and luciferase activity was analysed using a dual luciferase assay (Promega) in a Mithras LB 940 Multimode Microplate Reader (Berthold Technologies). Significance for assays was calculated using an unpaired *t* test in GraphPad Prism 5. Error bars are calculated as standard error of the mean (SEM).

For split TEV recruitment assays in a dose–response format, the following amendments to the general protocol were made. For a dose–response assay, all cells on the plate were transfected with the same receptor-NTEV-tcs-GV and adapter-CTEV fusions, the Fluc reporter plasmid, 1 ng of a plasmid constitutively expressing *Renilla* luciferase driven by a thymidine kinase promoter, and the EYFPnuc expressing plasmid. *Renilla* luciferase was used to assess toxic effects when applying compounds. For dose-dependent stimulation testing activation, six wells per condition were stimulated using increasing concentrations of an agonist at a semi-logarithmic scale. For assays testing inhibition, cells were treated with increasing concentrations of an antagonist at a semi-logarithmic scale, followed 1 h later by the addition of an agonist at a constant concentration. The following antagonists were used: lapatinib (Selleckchem), WZ4002 (Sigma-Aldrich), linsitinib (Selleckchem), and foretinib (Selleckchem). Dose–response data were analysed using the R-based ‘drc’ package, as described before [18, 19]. Error bars are calculated as standard error of the mean (SEM). For ease of presentation and intuition, we have transformed conventionally used IC_50_ values into pIC_50_ values [20]. These are calculated from IC_50_ values using the formula pIC_50_ = − log10(IC_50_), with units of molar for IC_50_ and therefore log(molar) for pIC_50_.

### Live cell split TEV recruitment assay

To identify an optimal time point of lysis for split TEV recruitment assays, firefly luciferase expression was continuously monitored using a 32-channel luminometer (lumicycler 32 by ActiMetrics) for 69 h starting from the starvation phase. For one assay, 1,000,000 PC12 cells were seeded on a 3.5 cm dish suitable for the instrument. The next day, 100 ng of receptor-NTEV-tcs-GV, 100 ng of adapter-CTEV, and 100 ng of Fluc reporter plasmids were transfected using 3.5 µl Lipofectamine 2000 and 1 ml Opti-MEM per dish. On day 2, cells were starved, and the cell culture medium was supplemented with 0.1% luciferin (Promega) to monitor firefly luciferase activity in a live cell setup. The dishes were wrapped with parafilm to avoid excess evaporation of medium and put into the luminometer, which was placed inside a cell culture incubator set to 37 °C and 5% CO_2_. All assays were run using three replicates per condition.

## Results

### Design of concatenated SH2 domains as universal adapter for split TEV recruitment assays

**Fig. 1:**
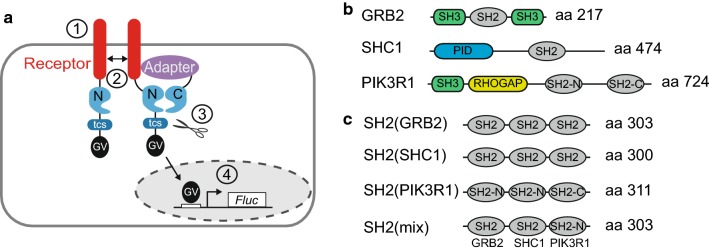
Design of a versatile split TEV recruitment assay for receptor tyrosine kinases. **a** Scheme of the split TEV recruitment assay for receptor tyrosine kinases (RTKs). RTKs are fused to an NTEV moiety along with a TEV protease cleavage site (tcs) and an artificial co-transcriptional activator GAL4-VP16 (GV). Adapter proteins are fused to CTEV. Upon activation by a specific ligand (1), the RTK dimerises, is cross-phosphorylated by the kinase domains at Tyr residues, providing docking sites for adapter proteins that bind to phosphorylated tyrosines (2). The ligand-induced interaction between RTK and adapter causes the NTEV and CTEV moieties to form a reconstituted TEV protease (2). Reconstituted TEV protease cleaves at tcs to release GV (3). Liberated GV migrates to the nucleus and initiates expression of firefly luciferase (Fluc) (4). **b** Domain organisation of full-length adapter proteins that are recruited by ERBB receptors. *SH2* src homology 2 domain, *SH3* src homology 3 domain, *PID* phosphotyrosine interaction domain, *RHOGAP* RhoGAP domain. Note that the adapter PIK3R1 contains two SH2 domains denoted as SH2-N (N-terminal) and SH2-C (C-terminal). **c** Domain organisation of the artificially concatenated SH2 domain phospho-adapters. For each clustered SH2 adapter, three single SH2 domains were fused. The SH2(mix) adapter contains an SH2 domain taken from each full-length adapter depicted in (**b**)

Split TEV assays are based on a TEV protease split into two inactive fragments, an N-terminal NTEV moiety and a C-terminal CTEV moiety. When assessing receptor activities, like for RTKs, the receptor is fused to the NTEV moiety, a TEV cleavage site (tcs), and the artificial co-activator GAL4-VP16 (GV), forming an NTEV-tcs-GV tag. Adapters are fused to CTEV (Fig. 1a). To establish RTK split TEV recruitment assays that use a universal adapter and are sensitive, robust, and likely reduce interference with cellular signalling as compared to native adapters, we designed artificial adapter proteins that only consist of clustered SH2 domains. The SH2 domain sequences were taken from the human adapter proteins GRB2, SHC1, and PIK3R1 (Fig. 1b). Notably, these adapters are known to interact with various RTK subfamilies, including the ERBB family [2], the INSR family [21], and the HGFR family [22]. For the ERBB family, we had previously developed split TEV recruitment assays using full-length PIK3R1, GRB2, and SHC1 as adapter proteins [14]. By contrast, each artificial adapter protein was constructed to contain three concatenated SH2 domains (Fig. 1c, Fig. S1). For artificial GRB2 and SHC1 adapters, the single SH2 domain present in the full protein was concatenated three times, termed SH2(GRB2) and SH2(SHC1). PIK3R1 contains two SH2 domains, an N-terminal SH2 domain (SH2-N), and a C-terminal SH2 domain (SH2-C). For the artificial adapter, two concatenated SH2-N domains were linked to a single SH2-C domain, termed SH2(PIK3R1). We also designed a chimeric protein adapter molecule containing one SH2 domain of each GRB2, SHC1, and the N-terminal SH2 domain of PIK3R1, termed SH2(mix). SH2 domains were separated via a flexible GS-linker formed of glycine, serine, and threonine residues (GGGGSTGGGGS) to allow for optimal folding and flexibility of binding.

### The concatenated SH2(GRB2) domain is a universal adapter for RTK split TEV recruitment assays

**Fig. 2:**
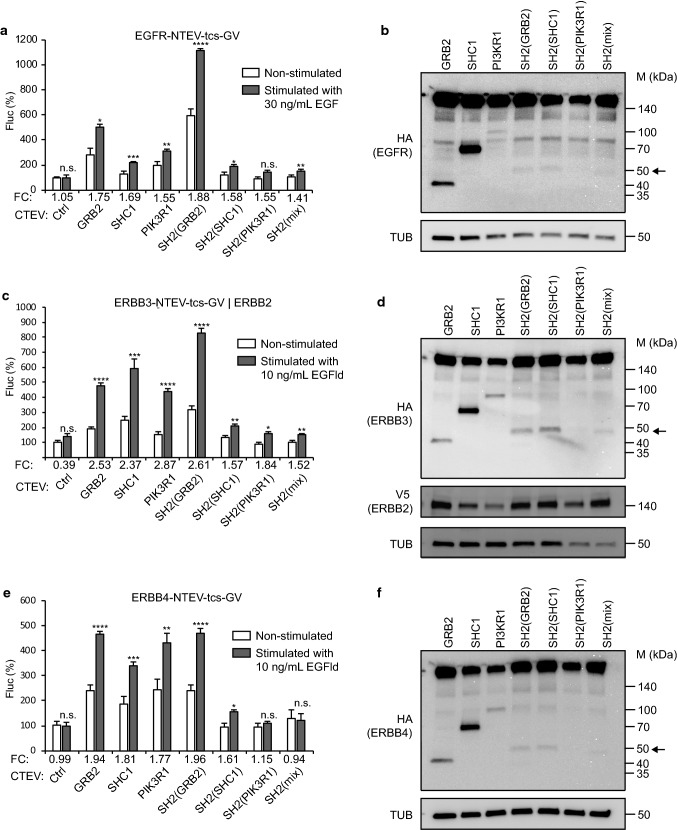
Comparing adapter protein performance for split TEV recruitment assays to monitoring ERBB receptor activities. Split TEV recruitment assays for ERBB family receptors. EGFR (**a**), ERBB2/ERBB3 (**c**), and ERBB4 (**e**) activities were assessed in PC12 cells using EGF to stimulate EGFR, and EGF-like domain (EGFld) to stimulate ERBB3 and ERBB4. For split TEV assays, the indicated receptor fusions were transfected together with indicated adapters that were fused to the CTEV moiety. Note that for the ERBB2/ERBB3 assay (**c**), ERBB2 is co-transfected to allow heterodimerisation and thus ERBB3 phosphorylation, which is required for the recruitment of adapters. Assays were stimulated for 16 h and analysed by a firefly luciferase assay. Non-stimulated samples are shown as open bars and stimulated ones as grey bars. *FC* fold change, *Ctrl* control (no adapter transfected). Results are shown as average of six samples, and error bars are shown as SEM. Significance was calculated using the unpaired *t* test, with **p ≤ 0.01; ***p ≤ 0.001; ****p ≤ 0.0001; *n.s.* not significant. Precise *p* values are provided in Table S1. Biochemical validation of the expression of ERBB receptors and adapters. Plasmids encoding EGFR (**b**), ERBB3 (**d**), and ERBB4 (**f**) (all tagged with NTEV-tcs-GV-2HA), ERBB2-V5 (**d**), and adapter proteins (all tagged with CTEV-2HA) were transiently transfected into PC12 cells, allowed to express for 16 h, and lysed. Lysates were subjected to Western blotting using the indicated antibodies. Calculated sizes of fusion proteins are provided in Table S1. Arrow indicates bands of artificial adapter fusions. Note that SH2(PIK3R1) is only very weakly expressed

For RTK split TEV recruitment assays, receptors were fused to the NTEV moiety along with tcs and GV, yielding RTK-NTEV-tcs-GV fusion proteins. As receptors, we selected EGFR, ERBB3, and ERBB4 of the ERBB family, IGF1R of the INSR family and MET of the HGFR family. Adapter proteins were fused to the CTEV moiety. HTS-compatible split TEV recruitment assays are performed using an end-point format (Fig. S2). Therefore, we first evaluated the optimal time point for this type of a split TEV assay. To do this, we monitored luciferase activity in a live cell split TEV recruitment assay using ERBB4 and PIK3R1, which has been used before in a compound screen [16]. ERBB4-NTEV-tcs-GV was transfected together with PIK3R1-CTEV and the Fluc reporter into PC12 cells, which were starved to reduce baseline activity, and thus enable proper stimulation by EGFld. The best stimulation to baseline ratio was obtained 16 h after stimulation (Fig. S3). Hence, all RTK split TEV recruitment assays using an end-point format were performed accordingly. To obtain a most sensitive adapter for RTK split TEV recruitment assays, we compared the performance of established full-length adapters versus artificial domain adapters. First, we monitored the induced activity of EGFR, ERBB3 (as heterodimerisation with ERBB2), and ERBB4 using the three full-length adapters GRB2, SHC1, and PIK3R1, as well as the SH2 domain adapters SH2(GRB2), SH2(SHC1), SH2(PIK3R1), and SH2(mix) in PC12 cells (Fig. 2, Table S1). In these assays, EGFR activity was stimulated using EGF, whereas ERBB3 and ERBB4 activity was stimulated using EGFld. Notably, fold changes using the SH2(GRB2) domain adapter scored highest for all ERBB receptor assays tested. Constitutive control *Renilla* luciferase readings remained stable for these assays (Fig. S4). In addition, various non-titrated amounts of transfected adapter plasmids that resulted in different expression lead to similar activation profiles of receptors, indicating that split TEV recruitment assays are robust and tolerate substantial differences in transfected adapter plasmids (Fig. S5). A live cell split TEV recruitment assay using ERBB4 and the SH2(GRB2) domain showed comparable kinetics to the ERBB4/PIK3R1 assay, indicating that the readout is stable over several hours (Fig. S3).

**Fig. 3:**
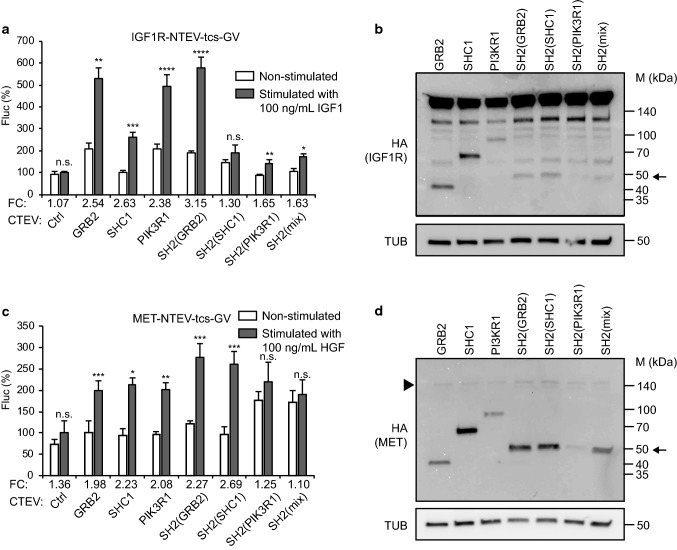
Comparing adapter protein performance for split TEV recruitment assays to monitoring IGF1R and MET receptor activities. **a**, **c** Split TEV recruitment assays for IGF1R and MET receptors. IGF1R (**a**) and MET (**c**) activities were assessed in PC12 cells using IGF1 to stimulate IGF1R, and HGF to stimulate MET. For split TEV assays, the indicated receptor fusions were transfected together with indicated adapters that were fused to the CTEV moiety. Assays were stimulated for 16 h and analysed by a firefly luciferase assay. Non-stimulated samples are shown as open bars and stimulated ones as grey bars. *FC* fold change, *Ctrl* control (no adapter transfected). Results are shown as average of six samples, error bars are shown as SEM. Significance was calculated using the unpaired *t* test, with **p ≤ 0.01; ***p ≤ 0.001; ****p ≤ 0.0001; *n.s.* not significant. Precise *p* values are provided in Table S1. **b**, **d** Biochemical validation of the expression of IGF1R and MET receptors and adapters. Plasmids encoding IGFR1 (**b**), and MET (**d**) (all tagged with NTEV-tcs-GV-2HA) and adapter proteins (all tagged with CTEV-2HA) were transiently transfected into PC12 cells, allowed to express for 16 h, and lysed. Lysates were subjected to Western blotting using the indicated antibodies. Calculated sizes of fusion proteins are provided in Table S1. Arrow indicates bands of artificial adapter fusions. Note that SH2(PIK3R1) is only very weakly expressed. Arrowhead indicates band for MET, which is also very weakly expressed

Then, we assessed whether RTK split TEV recruitment assays using full-length adapters and SH2 domain adapters can also be used to monitor the activity of RTKs that belong to other subfamilies. To do this, we selected IGF1R and MET that belong to INSR and HGFR families, respectively. Indeed, activation of IGF1R (using the ligand IGF1) and MET (using the ligand HGF) was robustly monitored using the SH2(GRB2) domain adapter, suggesting that the artificial adapter formed of three SH2(GRB2) domains serves as a universal adapter in split TEV recruitment assays (Fig. 3, Fig. S4). A Western blot analysis validates that the RTK-NTEV-tcs-GV fusion proteins for EGFR, ERBB3, ERBB4, IGF1R and MET (each harbouring an HA tag at the C-terminus) and all adapter-CTEV fusion proteins (each also harbouring an HA tag at the C-terminus) were correctly expressed at expected sizes in PC12 cells (Figs. 2b, d, f, 3b, d, Table S2). For the ERBB3 split TEV recruitment assay, a non-TEV-tagged, but V5-tagged ERBB2 was co-transfected to enable ligand-induced phosphorylation of ERBB3, and successively, formation of p-Tyr docking sites (Fig. 2d). Using immunocytochemistry, the proper expression of the RTK-NTEV-tcs-GV fusions was corroborated, as all RTK fusion proteins were enriched at the cell surface in PC12 cells (Fig. S6).

**Fig. 4:**
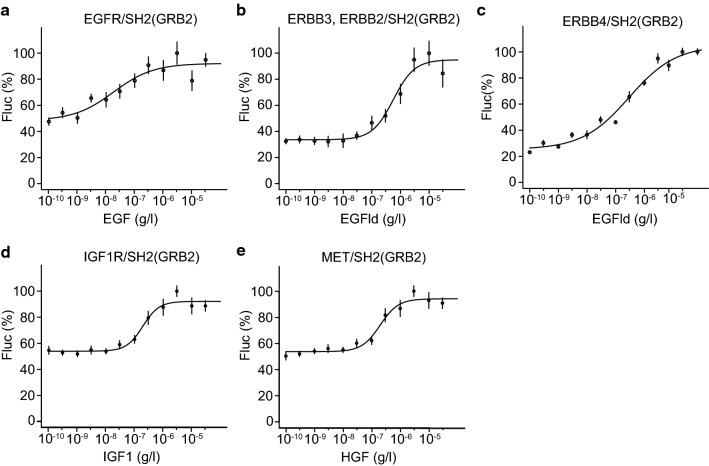
The concatenated SH2(GRB2) domain fusion is a universal adapter to profile ERBB, IGF1R, and MET activities. Split TEV recruitment assays using increasing concentrations of agonists (EGF, EGFld, IGF1 and HGF shown below *x* axis) for the receptors EGFR (**a**), ERBB2/ERBB3 (**b**), ERBB4 (**c**), IGF1R (**d**), and MET (**e**). Each receptor fusion (NTEV-tcs-GV tag for EGFR, ERBB3, ERBB4, IGF1R, MET; V5-tagged ERBB2) plasmid was co-transfected with the SH2(GRB2)-CTEV adapter plasmid into PC12 cells. Error bars are shown as SEM, with six replicates per condition

Next, we validated the sensitivity of RTK split TEV recruitment assays using the SH2(GRB2) adapter in agonist dose–response assays using increasing concentrations of the respective agonists (Fig. 4, Fig. S7). For ERBB recruitment assays, the SH2(SHC1) and SH2(mix) adapters showed, compared to the SH2(GRB2) adapter, lower signal-to-noise ratios and reduced sensitivity in dose–response assays (Fig. 4a–c, Figs. S7, S8). Notably, agonist dose–response split TEV recruitment assays for both IGF1R and MET and applying the SH2(GRB2) adapter also resulted in robust dose-dependent increases of receptor activity (Fig. 4d, e, Fig. S7). Taken together, we identified the three times concatenated SH2 domain of GRB2, termed SH2(GRB2), as universal adapter for our set of selected RTK receptor split TEV recruitment assays.

### The SH2(GRB2) universal adapter efficiently monitors lapatinib inhibition across all ERBB family receptors

**Fig. 5:**
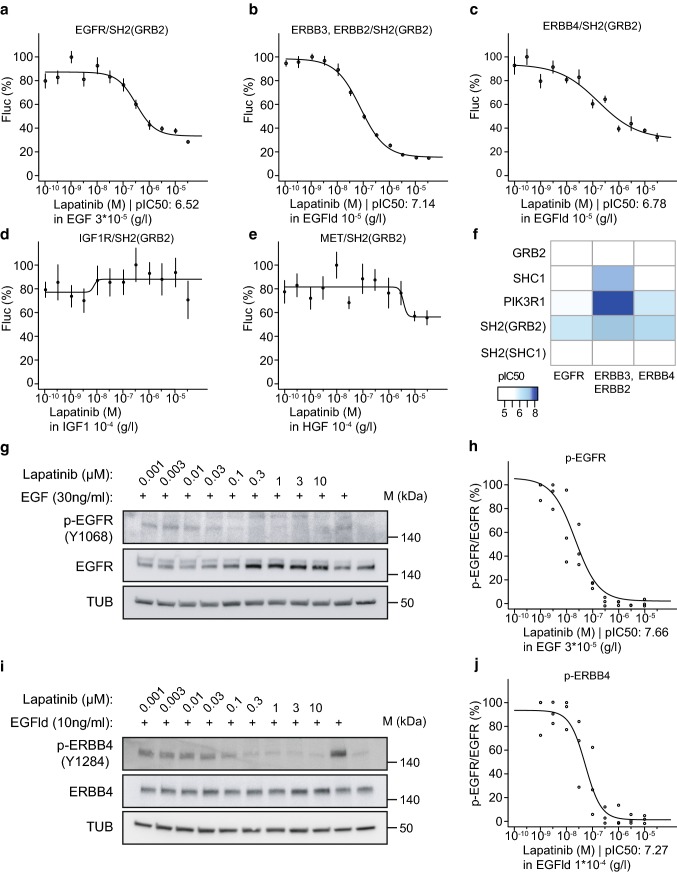
The universal SH2(GRB2) adapter displays the highest sensitivity to ERBB family inhibition by lapatinib. Split TEV recruitment assays monitoring the lapatinib-mediated inhibition of EGFR (**a**), ERBB2/ERBB3 (**b**), ERBB4 (**c**), IGF1R (**d**), and MET (**e**). Each receptor fusion (NTEV-tcs-GV tag for EGFR, ERBB3, ERBB4, IGF1R, MET; V5-tagged ERBB2) plasmid is co-transfected with the universal SH2(GRB2)-CTEV adapter plasmid into PC12 cells. Depicted are dose–response curves with a constant agonist stimulus (EGF, EGFld, IGF1, and HGF) and increasing concentrations of lapatinib. Error bars are shown as SEM, with six replicates per condition. **f** Heatmap displaying pIC_50_ values for lapatinib comparing assay performance of full-length and SH2(GRB2) domain concatenated CTEV adapters co-transfected with the ERBB family NTEV-tcs-GV fusions. Lapatinib reduces p-EGFR (Y1068) levels in A549 cells (**g**, **h**) and p-ERBB4 (Y1284) in T-47D cells (**i**, **j**). Cells were treated for 1 h with increasing concentrations of lapatinib and stimulated for 5 min with 30 ng/ml EGF (**g**) or 10 ng/ml EGFld (**i**) where indicated. Lysates were subjected to Western blotting and probed with indicated antibodies. Quantification of p-EGFR/EGFR levels (**h**) as shown in (**g**) and p-ERBB4/ERBB4 levels (**j**) as shown in (**i**) are plotted as dose–response curves. For each concentration depicted, three data points from three different lysates were used for calculations (c.f. Fig. S11a, b)

Cell-based assays are frequently used to assess a compound’s potential to inhibit the activity of a given receptor, such as for an RTK [23]. To measure the impact of inhibitory compounds on RTK targets using our split TEV recruitment assays, we challenged them with the well-characterised pan-ERBB family inhibitor lapatinib that is used in the clinic [24] and determined IC_50_ values for each assay (Fig. 5a–f, Figs. S9, S10). For a more intuitive comparison among assays, we transformed IC_50_ values into pIC_50_ values reflecting a logarithmic scale (see “Materials and methods” for details) (Fig. 5f, Table S3). When compared across ERBB receptor assays using both full-length and SH2 domain adapters, split TEV recruitment assays using the universal adapter SH2(GRB2) are most sensitive to lapatinib inhibition (Fig. 5f). The constitutive *Renilla* luciferase readout enabled us to discriminate between inhibitory and toxic effects, with the latter ones only occurring at 30 µM lapatinib (Fig. S9). We found in our assays using the SH2(GRB2) adapter that lapatinib efficiently inhibits EGFR (IC_50_: 305 nM, pIC_50_: 6.52) and ERBB2/ERBB3 (IC_50_: 72 nM, pIC_50_: 7.14), and ERBB4 (IC_50_: 166 nM, pIC_50_: 6.78). However, lapatinib treatment did not inhibit IGF1R and MET activities (Fig. 5d, e, Fig. S9). In concordance with the literature, stimulated IGF1R and MET receptors were efficiently inhibited by linsitinib and foretinib, respectively (Fig. S9) [25, 26]. To further compare the sensitivity of our split TEV RTK recruitment assays with cellular assays that use phospho-specific antibodies to monitor RTK activity, we explored the potency of EGFR and ERBB4 inhibition by lapatinib by assessing the phosphorylation levels of EGFR in A549 cells and of ERBB4 in T-47D cells. Both cell lines are of human origin and reasonably express EGFR and ERBB4, respectively. Dose-dependent addition of lapatinib led to an efficient inhibition of p-EGFR and p-ERBB4, as revealed by Western blotting (Fig. 5g–j, Fig. S11). Quantification of our Western blotting data indicate that EGFR and ERBB4 are inhibited by lapatinib at similar concentrations in cellular assays when comparing antibody-based detection of phosphorylation levels and split TEV-based RTK recruitment assays. By contrast, a biochemical kinome profiling study reported that both EGFR (2.4 nM, kD) and ERBB2 (7 nM, kD) are more potently inhibited than ERBB4 (54 nM, kD) [27]. In support of our findings, data from published cellular assays, which use lysates as input for enzyme-linked immunosorbent assays (ELISA) and applied phospho-specific antibodies as sensor of receptor activity, reported a similar range of concentrations for IC_50_ values of EGFR and ERBB2 inhibition, suggesting that efficiencies may substantially vary between biochemical and cell-based assays (Table 1, Table S4) [24, 27–29]. Taken together, our own data obtained from the split TEV recruitment assays indicate that lapatinib efficiently inhibits ERBB receptors, with preferentially inhibiting ERBB2/ERBB3 (72 nM, IC_50_) and ERBB4 (166 nM, IC_50_) over EGFR (305 nM, IC_50_).

**Table 1:** IC_50_ and K_D_ values for ERBB receptor family inhibition by lapatinib and WZ4002 in cellular and biochemical assays

Name of compound	Compound ID (CID)	Target	PubChem assay ID (AID)	Type of assay	IC_50_ (nM)/*K*_D_	pIC_50_	References
Lapatinib	208908	EGFR	474116	ELISA, cellular lysates	52	7.28	[28]
Lapatinib	208908	EGFR	517323	ELISA, cellular lysates	433	6.36	[29]
Lapatinib	208908	EGFR	n.a.	Split TEV, cell-based assay	305	6.52	This study
Lapatinib	208908	EGFR	624996	Biochemical	2.4	8.62	[27]
Lapatinib	208908	ERBB2	474117	ELISA, cellular lysates	100	7.00	[28]
Lapatinib	208908	ERBB2	517324	ELISA, cellular lysates	140	6.85	[29]
Lapatinib	208908	ERBB2/ERBB3	n.a.	Split TEV, cell-based assay	72	7.14	This study
Lapatinib	208908	ERBB2	624804	Biochemical	7	8.15	[27]
Lapatinib	208908	ERBB3	624851	Biochemical	5500	5.26	[27]
Lapatinib	208908	ERBB4	n.a.	Split TEV, cell-based assay	166	6.78	This study
Lapatinib	208908	ERBB4	624815	Biochemical	54	7.27	[27]
WZ4002	44607530	EGFR	770081	Western blotting, cellular lysates	1180	5.93	[30]
WZ4002	44607530	EGFR	n.a.	Split TEV, cell-based assay	4019	5.40	This study
WZ4002	44607530	EGFR	1204628	Biochemical	16	7.79	[55]
WZ4002	44607530	ERBB2/ERBB3	n.a.	Split TEV, cell-based assay	215	6.67	This study
WZ4002	44607530	ERBB2	1204629	Biochemical	0.42	9.37	[55]
WZ4002	44607530	ERBB3	1204629	Biochemical	0.42	9.37	[55]
WZ4002	44607530	ERBB4	n.a.	Split TEV, cell-based assay	1007	6.00	This study
WZ4002	44607530	ERBB4	1204629	Biochemical	0.42	9.37	[55]

ELISA and Western blotting data were retrieved from the PubChem database (https://pubchem.ncbi.nlm.nih.gov) and indicated references. Note that ELISA and Western blotting data from these sources were obtained using phospho-specific antibodies and cellular lysates. The full data set comprising both biochemical and cellular assays are shown in Table S4 (lapatinib) and Table S5 (WZ4002)

*n.a.* not applicable

**Fig. 6:**
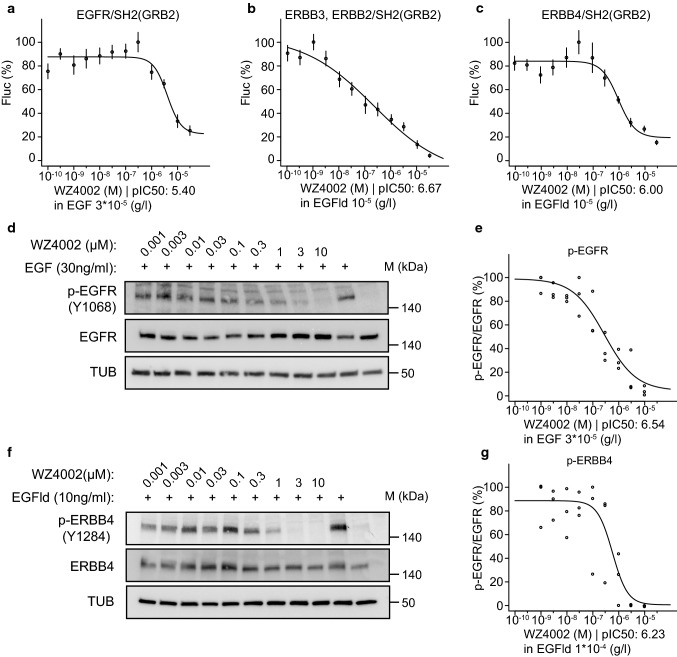
The ERBB family antagonist WZ4002 inhibits split TEV recruitment assays using the universal SH2(GRB2) adapter. Split TEV recruitment assays monitoring the WZ4002-mediated inhibition of EGFR (**a**), ERBB2/ERBB3 (**b**), and ERBB4 (**c**). Each receptor fusion (NTEV-tcs-GV tag for EGFR, ERBB3, ERBB4; V5-tagged ERBB2) plasmid is co-transfected with the universal SH2(GRB2) CTEV adapter into PC12 cells. Depicted are dose–response curves with a constant stimulus (EGF, EGFld) and increasing concentrations of lapatinib. Error bars are shown as SEM, with six replicates per condition. WZ4002 reduces p-EGFR (Y1068) levels in A549 cells (**d**, **e**) and p-ERBB4 (Y1284) in T-47D cells (**f**, **g**). Cells were treated for 1 h with increasing concentrations of WZ4002 and stimulated for 5 min with 30 ng/ml EGF (**d**) or 10 ng/ml EGFld (**f**) where indicated. Lysates were subjected to Western blotting and probed with the indicated antibodies. Quantification of p-EGFR/EGFR levels (**e**) as shown in (**d**) and p-ERBB4/ERBB4 levels (**g**) as shown in (**f**) are plotted as dose–response curves. For each concentration depicted, three data points from three different lysates were used for calculations (c.f. Fig. S11c, d)

To corroborate the use of our cell-based assays using the split TEV recruitment technique and the SH2(GRB2) adapter, we also tested the pan-ERBB inhibitor WZ4002 (Table 1, Table S5) [30, 31]. By performing antagonistic dose–response assays for WZ4002, we could confirm the usage of SH2(GRB2) as a universal adapter in ERBB split TEV recruitment assays. In split TEV assays, WZ4002 efficiently inhibited ERBB family receptors EGFR, ERBB3, and ERBB4 (Fig. 6a–c). Dose-dependent addition of WZ4002 to A549 and T-47D cells reduced phospho-levels of EGFR and ERBB4 to similar levels when compared to split TEV recruitment assays, as quantification of phospho-EGFR and phospho-ERBB4 indicates (Fig. 6d–g, Fig. S11). Further, we tested whether spironolactone exerts antagonistic effects on ERBB family receptors as recently reported [16]. Spironolactone is a pan-ERBB inhibitor, displaying selectivity for ERBB4 over EGFR, as previously determined using a split TEV dimerisation assay. Spironolactone also inhibited ERBB activities using the universal SH2(GRB2) adapter in the split TEV recruitment assays, with some selectivity for ERBB4 over EGFR (Fig. S12). Further, the spironolactone metabolite canrenone did not exhibit any antagonistic effects on any ERBB assay, which is also consistent with our previous findings [16] (Fig. S12). In summary, these data suggest that the adapter SH2(GRB2) can be used in split TEV recruitment assays to monitor both agonist and antagonist actions targeting the RTK ERBB family in living cells.

## Discussion

We describe a genetically encoded split TEV recruitment assay to monitor RTK activities using a universal adapter that consists of three concatenated SH2 domains, which bind to phosphorylated tyrosine residues. As examples for RTKs, we have selected the ERBB receptor family, IGF1R of the INSR family, and MET of the HGFR family. We tested full-length adapters GRB2, SHC1, and PIK3R1, as well as artificially constructed adapters consisting of concatenated SH2 domains of GRB2, SHC1, and PIK3R1 for efficient assay performance upon RTK receptor stimulation (Fig. 1). The adapter formed of three concatenated SH2 domains of GRB2, SH2(GRB2) displayed the best signal-to-noise ratio across all RTK biosensor assays tested (Figs. 2, 3), and the performance of this adapter was further validated in dose–response assays (Fig. 4). In addition, the SH2(GRB2) adapter was characterised in antagonist dose–response assays by applying the ERBB family inhibitors lapatinib, WZ4002, and spironolactone (Figs. 5, 6, Fig. S12).

### SH2(GRB2) as a universal adapter for RTK activity measurement

For integrating cell-based assays into HTS, an appropriate signal-to-noise ratio is key to robustness [23, 32]. Therefore, the SH2(GRB2) adapter represents a universal approach towards HTS assays. In agreement, the SH2 domain of GRB2 was reported to bind all members of the ERBB family in a biochemical study using protein microarrays, supporting our findings of SH2(GRB2) as universal adapter [33]. The SH2 domain of GRB2 was also applied as p-Tyr sensor in living cells, where the SH2 domain was fused to the photoactivatable fluorescent protein tdEos to monitor EGFR activation [34]. Furthermore, full-length GRB2 has been shown to stronger bind to p-Tyr motifs present in EGFR and ERBB4 when compared to SHC1, suggesting that the SH2 domain of GRB2 is a better fit for a universal adapter in split TEV recruitment assays [35]. The general consensus sequence of the p-Tyr motifs that the SH2 domain of GRB2 binds to was initially described as pY-X-N [36]. However, a more recent study expanded this view to a more general consensus sequence pY-[ϕ/Q]-[NQFDK], where ϕ stands for a hydrophobic residue [35], supporting the notion that the SH2 domain of GRB2 can bind to p-Tyr motifs with a rather flexible sequence. As the SH2 domain adapter only consists of SH2 domains and no other interaction modules are present in this adapter, this artificial adapter may solely function as a p-Tyr sensor [37]. Furthermore, the SH2(GRB2) adapter may be used for monitoring activities of other RTKs, such as the insulin growth factor receptor and tropomyosin receptor kinase families that also bind GRB2, potentially expanding the number of receptors [38, 39]. The split TEV recruitment assays presented are based on transient transfections and thus use overexpressed receptors and adapters. Therefore, we would like to emphasise that RTK split TEV recruitment assays were specifically designed to assay receptor activities in heterologous cells, and these assays may not be combined with analyses of downstream signalling [40]. As heterologous cells display abnormal activities of downstream signalling, events of cellular signalling should preferably be monitored in primary cell types. By contrast, activities of receptors may well be studied in heterologous cell lines, as studying receptor activities implicates the first step of a signalling cascade [23].

When RTKs become activated, the phosphorylation of intracellular tyrosine residues represents the first step upon ligand binding. Notably, different agonist ligands and varying concentrations thereof may cause diverse cellular outcomes, as for example described for EGFR ligands that differentially affect EGFR endocytosis and recycling [41]. Thus, it is of common interest to specify which of these tyrosine residues of an RTK are phosphorylated and act as docking sites for adapter proteins to initiate signalling [42]. Alternatively, phosphorylated docking sites may act additively to elicit a response by recruiting a defined set of adapters [43]. To understand which phospho-signature is generated by a given RTK, e.g. after agonist or antagonist treatment, full-length adapters as well as SH2 domain adapters may be used for profiling of biased adapter recruitment. In our split TEV recruitment assays, EGFR activation, for example, can be detected at very low EGF concentrations using the GRB2 full-length protein as adapter (Fig. S8). By contrast, when treating ERBB3 with the antagonist lapatinib, full-length PIK3R1 proved to be the most sensitive adapter protein to measure an inhibition (Fig. 5f, Fig. S10). The adapter SH2(GRB2) does not cover aspects of biased signalling per se, but can be used as universal adapter to study RTK activity profiles. Assessing differential binding properties may be important, as adapters have varying binding affinities to activated receptors and binding affinities depend on ligand concentrations [44]. Likewise, receptors can recruit distinct sets of adapter proteins to initiate specific downstream signalling [45]. Thus, the SH2(GRB2) adapter may be used for a primary assessment of RTK activity in recruitment assays, followed by more specialised assays (e.g. using other adaptors in split TEV recruitment assays or cell-based assays using phospho-antibodies) to determine signalling fate.

In addition, usage of the universal adapter is not restricted to split TEV-based recruitment assays, but can be implemented into all genetically encoded recruitment assays that, for example, rely on the complementation of a reporter protein, the release of an artificial transcription factor, or both. For example, split green fluorescent protein (GFP) assays (and derivatives) [46, 47], split firefly luciferase [48], split ubiquitin [49], and full-TEV protease assays [50] may be applicable. Furthermore, the SH2(GRB2) adapter may also be used in fluorescence resonance energy transfer (FRET) and bioluminescence resonance energy transfer (BRET)-based assays [51, 52]. Taken together, the universal SH2(GRB2) adapter represents a p-Tyr biosensor to monitor RTK activities and may be used in various cell-based assays that are genetically encoded.

### Lapatinib preferentially inhibits ERBB4 over EGFR in RTK/SH2 adapter recruitment assays

Lapatinib, an EGFR and ERBB2 receptor inhibitor, is used in the clinics, e.g. in combination therapies to treat cancers [53, 54]. It has been shown that lapatinib also inhibits ERBB3 and ERBB4 receptors in biochemical profiling studies [27]. Compared to data obtained from biochemical assays, IC_50_ concentrations for lapatinib were substantially higher in cell-based assays [24, 27, 28] (Table 1, Table S4), suggesting that efficiencies of compounds in living cells cannot be precisely predicted from biochemical data. Thus, compounds should be efficiently tested in cell-based assays that best reflect the nature or the target or, in the case of a disease-linked phenotype, most faithfully replicate the disease state [23]. Furthermore, antagonistic preferences of a given target over related targets, a feature important for characterising selectivity of a compound, may vary between biochemical and cell-based assays, supporting the notion of applying the most appropriate test system possible.

### Profiling multiple RTK activities simultaneously using multiplexed cell-based assays

The abnormal activities of RTKs are linked to the pathophysiology of various human diseases, such as cancers, diabetes, inflammation, angiogenesis, neurodegenerative diseases, and psychiatric disorders [2, 3]. Therefore, these associations have initiated the development of drugs that block or attenuate aberrant activity of RTKs. However, many available drugs targeting RTKs lack selectivity, demonstrating the medical need for the development of better drugs. The need for more specific drugs is also reflected by the fact that only 3% of all marketed drugs target kinases including RTKs [4]. Cell-based profiling techniques that enable the simultaneous analysis of multiple targets and allow defining selectivity of a given compound will contribute to the development of better drugs [12]. For example, multiplexed cell-based assays that rely on complementation of a reporter, the release of an artificial transcription factor, and the use of barcoded RNA sequences as reporters can be applied to profile activities of disease-relevant targets, as we have recently shown for G protein-coupled receptors (GPCRs) [40]. Therefore, using the split TEV recruitment assay and integrating the universal SH2(GRB2) adapter may represent a promising approach to build a technology platform to assess RTK activities in early drug discovery to finally improve compound selectivity.

## Electronic supplementary material

Below is the link to the electronic supplementary material.

Supplementary material 1 (PDF 2340 kb)

## Abbreviations

EGF: Epidermal growth factor
EGFld: EGF-like domain of NRG1
EGFR: Epidermal growth factor receptor
ERBB2: Erb-B2 receptor tyrosine kinase 2
ERBB3: Erb-B2 receptor tyrosine kinase 3
ERBB4: Erb-B2 receptor tyrosine kinase 4
GRB2: Growth factor receptor bound protein 2
HTS: High-throughput screening
IGF1R: Insulin growth factor 1 receptor
MET: Mesenchymal epithelial transition proto-oncogene, receptor tyrosine kinase
NRG1: Neuregulin 1
PIK3R1: Phosphoinositide-3-kinase regulatory subunit 1
RTK: Receptor tyrosine kinase
SHC1: Src homology 2 domain-containing adaptor protein 1
SH2: Src homology 2
TEV: Tobacco etch virus
