# Supplementary material for: Monitoring activities of receptor tyrosine kinases using a universal adapter in genetically encoded split TEV assays
Source: Cell Mol Life Sci. 2019 Jan 8;76(6):1185–99. doi: 10.1007/s00018-018-03003-2 (PMC6675780; doi:10.1007/s00018-018-03003-2)
Supplement: Supplementary file 1 — Supplementary material 1 (PDF 2340 kb) [file 18_2018_3003_MOESM1_ESM.pdf]

Electronic supplementary material

Monitoring activities of receptor tyrosine kinases using a universal adapter in genetically encoded split TEV assays

Jan P. Wintgens[1,2], Sven P. Wichert[1,2], Luksa Popovic[2], Moritz J. Rossner[1], Michael C. Wehr[1,2*]

(1) Department of Psychiatry and Psychotherapy, University Hospital, LMU Munich, Nussbaumstr. 7, 80336 Munich, Germany

(2) Systasy Bioscience GmbH, Adams-Lehmann-Str. 56, 80797 Munich, Germany

(*) Author for correspondence: Dr. Michael C. Wehr, email: Michael.Wehr@med.uni-muenchen.de; phone: +49 (0) 89 4400 53275

Supplementary Figures.. 3
Supplementary Tables 20

Supplementary Figures

a SH2(GRB2) – 3xSH2 of GRB2

DNA sequence:

atgGTGtggtttttggcaaaatccccagagccaaggcagaagaaatgcttagcaaacagcggcacgatggggcc
tttcttatccgagagagtgagagcgctcctggggacttctccctctctgtcaagtttggaaacgatgtgcagcac
ttcaaggtgctccgagatggagccgggaagtacttcctctgggtggtgaagttcaattctttgaatgagctggtg
gattatcacagatctacatctgtctccagaaaccagcagatattcctgcgggacatagaaGGAGGTGGAGGTAGC
ACCGGTGGAGGAGGTAGCtggtttttggcaaaatccccagagccaaggcagaagaaatgcttagcaaacagcgg
cacgatggggcctttcttatccgagagagtgagagcgctcctggggacttctccctctctgtcaagtttggaaac
gatgtgcagcacttcaaggtgctccgagatggagccgggaagtacttcctctgggtggtgaagttcaattctttg
aatgagctggtggattatcacagatctacatctgtctccagaaaccagcagatattcctgcgggacatagaaGGA
GGTGGAGGTAGCACCGGTGGAGGAGGTAGCtggtttttggcaaaatccccagagccaaggcagaagaaatgctt
agcaaacagcggcacgatggggcctttcttatccgagagagtgagagcgctcctggggacttctccctctctgtc
aagtttggaaacgatgtgcagcacttcaaggtgctccgagatggagccgggaagtacttcctctgggtggtgaag
ttcaattctttgaatgagctggtggattatcacagatctacatctgtctccagaaaccagcagatattcctgcgg
gacatagaa

Protein sequence:

MVWFFGKIPRAKAEEMLSKQRHDGAFLIRESESAPGDFSLSVKFGNDVQHFKVLRDGAGKYFLWVVKFNSLNELV
DYHRSTSVSRNQQIFLRDIEGGGGSTGGGGSWFFGKIPRAKAEEMLSKQRHDGAFLIRESESAPGDFSLSVKFGN
DVQHFKVLRDGAGKYFLWVVKFNSLNELVDYHRSTSVSRNQQIFLRDIEGGGGSTGGGGSWFFGKIPRAKAEEML
SKQRHDGAFLIRESESAPGDFSLSVKFGNDVQHFKVLRDGAGKYFLWVVKFNSLNELVDYHRSTSVSRNQQIFLR
DIE

b SH2(SHC1) – 3xSH2 of SHC1

DNA sequence:

atgGTGtggttccatgggaagctgagccggcgggaggctgaggcactgctgcagctcaatggggacttcctggta
cgggagagcacgaccacacctggccagtatgtgctcactggcttgcagagtgggcagcctaagcatttgctactg
gtggaccctgagggtgtggttcggactaaggatcaccgctttgaaagtgtcagtcaccttatcagctaccacatg
gacaatcacttgcccatcatctctgcgggcagcgaactgtgtctacagcaacctgtgGGAGGTGGAGGTAGCACC
GGTGGAGGAGGTAGCtggttccatgggaagctgagccggcgggaggctgaggcactgctgcagctcaatggggac
ttcctggtacgggagagcacgaccacacctggccagtatgtgctcactggcttgcagagtgggcagcctaagcat
ttgctactggtggaccctgagggtgtggttcggactaaggatcaccgctttgaaagtgtcagtcaccttatcagc
taccacatggacaatcacttgcccatcatctctgcgggcagcgaactgtgtctacagcaacctgtgGGAGGTGGA
GGTAGCACCGGTGGAGGAGGTAGCtggttccatgggaagctgagccggcgggaggctgaggcactgctgcagctc
aatggggacttcctggtacgggagagcacgaccacacctggccagtatgtgctcactggcttgcagagtgggcag
cctaagcatttgctactggtggaccctgagggtgtggttcggactaaggatcaccgctttgaaagtgtcagtcac
cttatcagctaccacatggacaatcacttgcccatcatctctgcgggcagcgaactgtgtctacagcaacctgtg

Protein sequence:

MVWFHGKLSRREAEALLQLNGDFLVRESTTTPGQYVLTGLQSGQPKHLLLVDPEGVVRTKDHRFESVSHLISYHM
DNHLPIISAGSELCLQQPVGGGGSTGGGGSWFHGKLSRREAEALLQLNGDFLVRESTTTPGQYVLTGLQSGQPKH
LLLVDPEGVVRTKDHRFESVSHLISYHMDNHLPIISAGSELCLQQPVGGGGSTGGGGSWFHGKLSRREAEALLQL
NGDFLVRESTTTPGQYVLTGLQSGQPKHLLLVDPEGVVRTKDHRFESVSHLISYHMDNHLPIISAGSELCLQQPV

Fig. S1 continued

c SH2(PIK3R1) – 3xSH2 of PIK3R1(SH2a, SH2a, SH2b)

DNA sequence:

atgGTGtggtactggggagatatctcgagggaagaagtgaatgaaaaacttcgagatacagcagacgggacctttt
ttggtacgagatgcgtctactaaaatgcatggtgattatactcttacactaaggaaaggggaaataacaatta
atcaaaatatttcatcgagatgggaaatatggcttctctgacccattaaccttcagttctgtggttgaattaata
aaccactaccggaatgaatctctagctcagtataatcccaaattggatgtgaaattactttatccagtaGGAGGT
GGAGGTAGCACCGGTGGAGGAGGTAGCtggtactggggagatatctcgagggaagaagtgaatgaaaaacttcga
gatacagcagacgggaccttttttggtacgagatgcgtctactaaaatgcatggtgattatactcttacactaagg
aaaggggaaataacaaattaatcaaaatatttcatcgagatgggaaatatggcttctctgacccattaaccttc
agttctgtggttgaattaataaaccactaccggaatgaatctctagctcagtataatcccaaattggatgtgaaa
ttactttatccagtaGGAGGTGGAGGTAGCACCGGTGGAGGAGGTAGCtggaatgttggaagcagcaaccgaaac
aaagctgaaaacctgttgcgagggaagcgagatggcacttttcttgtccgggagagcagtaaacagggctgctat
gcctgctctgtagtggtggacggcgaagtaaagcattgtgtcataaacaaaacagcaactggctatggctttgcc
gagccctataacttgtacagctctctgaaagaactggtgctacattaccaacacacctcccttgtgcagcacaac
gactccctcaatgtcacactagcctacccagta

Protein sequence:

MVWYWGDISREEVNEKLRDTADGTFLVRDASTKMHGDYTLTLRKGGNNKLIKIFHRDGKYGFSDPLTFSSVVELI
NHYRNESLAQYNPKLDVKLLYPVGGGGSTGGGGSWYWGDISREEVNEKLRDTADGTFLVRDASTKMHGDYTLTLR
KGGNNKLIKIFHRDGKYGFSDPLTFSSVVELINHYRNESLAQYNPKLDVKLLYPVGGGGSTGGGGSWNVGSSNRN
KAENLLRGKRDGTFLVRESSKQGCYACSVVVDGEVKHCVINKTATGYGFAEPYNLYSSLKELVLHYQHTSLVQHN
DSLNVTLAYPV

d SH2(mix) – 1xSH2 of GRB2, 1xSH2 of SHC1, 1xSH2a of PIK3R1

DNA sequence:

atgGTGtggtttttggcaaaatccccagagccaaggcagaagaaatgcttagcaaacagcggcacgatggggcc
tttcttatccgagagagtgagagcgctcctggggacttctccctctctgtcaagtttggaaacgatgtgcagcac
ttcaaggtgctccgagatggagccgggaagtacttcctctgggtggtgaagttcaattctttgaatgagctggtg
gattatcacagatctacatctgtctccagaaaccagcagatattcctgcgggacatagaaGGAGGTGGAGGTAGC
GGAGGTGGAGGTAGCtggttccatgggaagctgagccggcgggaggctgaggcactgctgcagctcaatggggac
ttcctggtacgggagagcacgaccacacctggccagtatgtgctcactggcttgcagagtgggcagcctaagcat
ttgctactggtggaccctgagggtgtggttcggactaaggatcaccgctttgaaagtgtcagtcaccttatcagc
taccacatggacaatcacttgcccatcatctctgcgggcagcgaactgtgtctacagcaacctgtgGGAGGTGGA
GGTAGCGGAGGTGGAGGTAGCtggtactggggagatatctcgagggaagaagtgaatgaaaaacttcgagataca
gcagacgggaccttttttggtacgagatgcgtctactaaaatgcatggtgattatactcttacactaaggaaaggg
ggaaataacaaattaatcaaaatatttcatcgagatgggaaatatggcttctctgacccattaaccttcagttct
gtggttgaattaataaaccactaccggaatgaatctctagctcagtataatcccaaattggatgtgaaattactt
tatccagta

Protein sequence:

MVWFFGKIPRAKAEEMLSKQRHDGAFLIRESESAPGDFSLSVKFGNDVQHFKVLRDGAGKYFLWVVKFNSLNELV
DYHRSTSVSRNQQIFLRDIEGGGGSGGGGSWFHGKLSRREAEALLQLNGDFLVRESTTTPGQYVLTGLQSGQPKH
LLLVDPEGVVRTKDHRFESVSHLISYHMDNHLPIISAGSELCLQQPVGGGGSGGGGSWYWGDISREEVNEKLRDT
ADGTFLVRDASTKMHGDYTLTLRKGGNNKLIKIFHRDGKYGFSDPLTFSSVVELINHYRNESLAQYNPKLDVKLL
YPV

Fig. S1. DNA and protein sequences of concatenated SH2 domain adapters.

SH2 domains are highlighted in cyan (for DNA sequences) and green (for protein sequences). SH2 domains are spaced by flexible linkers (with protein sequence GGGGSTGGGGS). (**a**) Three concatenated SH2 domains of human GRB2; denoted as SH2(GRB2). (**b**) Three concatenated SH2 domains of human SHC1; denoted as SH2(SHC1). (**c**) Three concatenated SH2 domains of human PIK3R1, using twice the N-terminal SH2 domain (SH2-N) of PIK3R1, and once the C-terminal SH2 domain (SH2-C) of PIK3R1; denoted as SH2(PIK3R1). (**d**) Three concatenated SH2 domains of human GRB2, human SHC1, and the N-terminal SH2 domain (SH2-N) of human PIK3R1; denoted as SH2(mix).

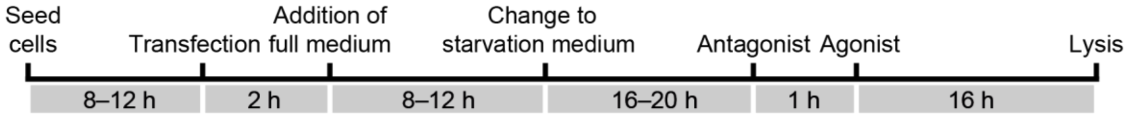

Fig. S2. Experimental time line for agonist and antagonist ERBB split TEV recruitment assays.

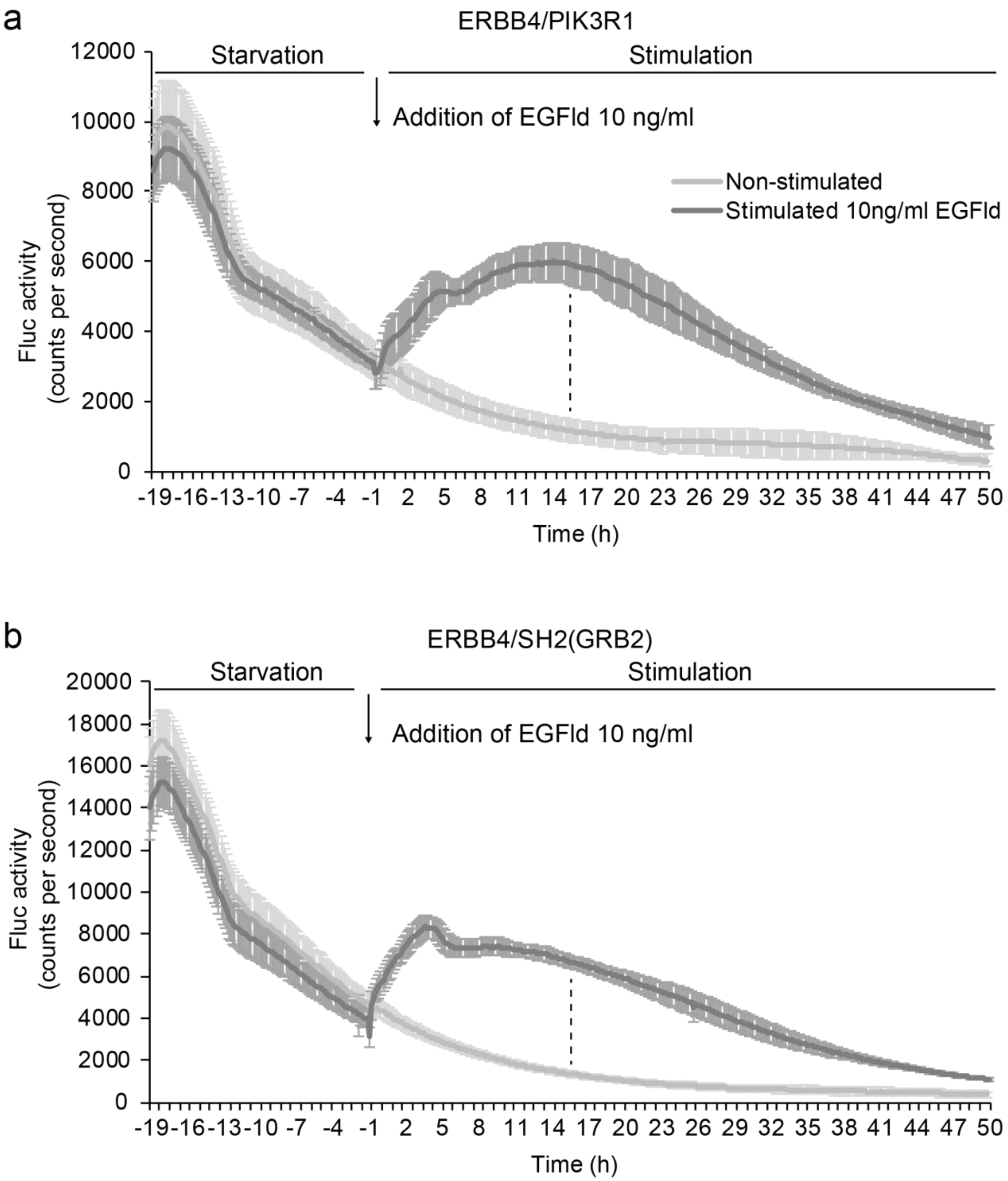

Fig. S3. Split TEV ERBB4 recruitment assays optimally respond after 16 h of stimulation.

The ERBB4-NTEV-tcs-GV (ERBB4) receptor plasmid was transfected into PC12 cells together with a firefly luciferase (Fluc) reporter plasmid, and either a plasmid encoding the full-length adapter PIK3R1-CTEV (assay: ERBB4/PIK3R1) (**a**) or the SH2-domain adapter SH2(GRB2)-CTEV (assay: ERBB4/SH2(GRB2) (**b**). Luciferase activity was measured every 10 minutes. Time point at 0 h denotes the start of the stimulation phase at which 10 ng/mL EGF-like domain (EGFld) were added to the medium. The dashed line at 16 h represents the highest ratio of stimulation to baseline activity. An average of three samples is shown, error bars represent SD.

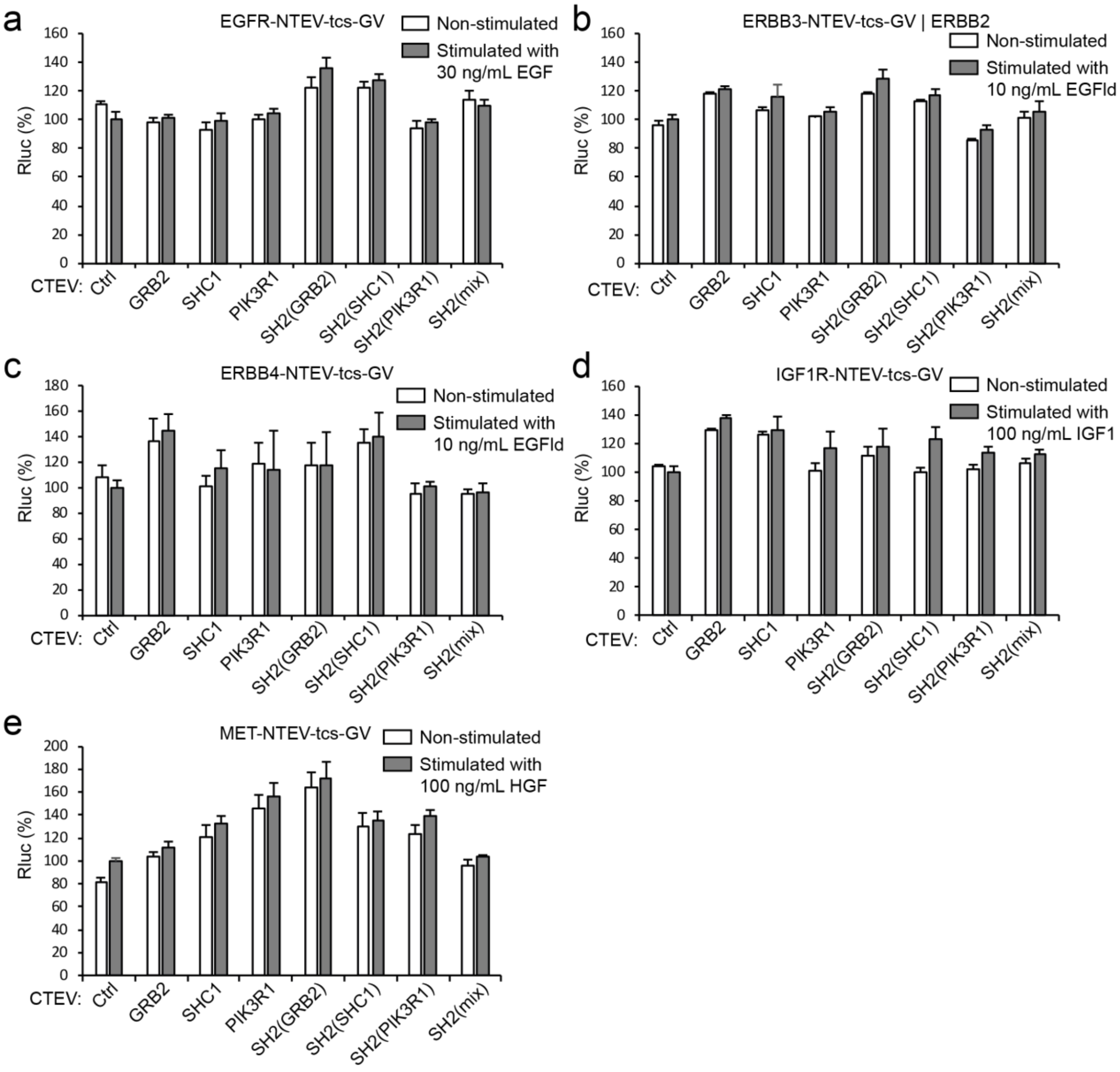

Fig. S4. Constitutive *Renilla* luciferase readings are stable in growth factor stimulated RTK split TEV recruitment assays.

Thymidine kinase-driven *Renilla* luciferase signals were assessed in EGFR (**a**), ERBB2/ERBB3 (**b**), ERBB4 (**c**), IGF1R (**d**), and MET (**e**) split TEV recruitment assays under control and stimulated conditions in PC12 cells. EGF was applied to stimulate EGFR, EGF-like domain (EGFld) to stimulate ERBB3 and ERBB4, IGF1 to stimulate IGF1R and HGF to stimulate MET. The indicated receptor fusions were transfected together with indicated adapters that were fused to the CTEV moiety. Note that for the ERBB2/ERBB3 assay (**b**), ERBB2 is co-transfected to allow heterodimerisation and thus ERBB3 phosphorylation, which is required for the recruitment of adapters. Assays were stimulated for 16 h, lysed and analysed for *Renilla* activity. Non-stimulated samples are shown as open bars and stimulated once as grey bars. Error bars represent SD, with 6 replicates per condition. Fig. S5 correlates to Figs. 2 and 3.

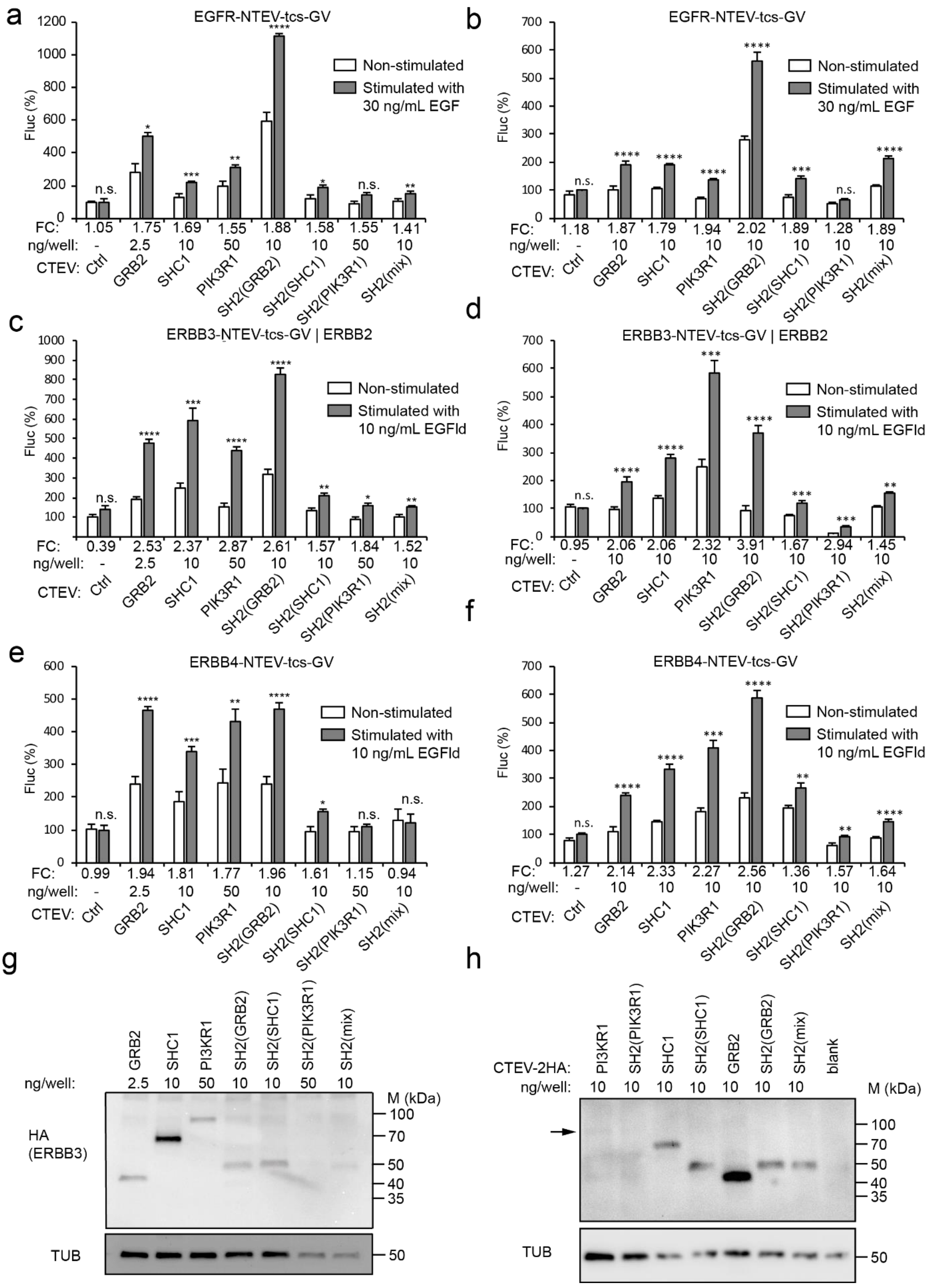
a
EGFR-NTEV-tcs-GV
Fluc (%)
Non-stimulated
Stimulated with 30 ng/mL EGF
FC: 1.05 1.75 1.69 1.55 1.88 1.58 1.55 1.41
ng/well: - 2.5 10 50 10 10 50 10
CTEV: Ctrl GRB2 SHC1 PIK3R1 SH2(GRB2) SH2(SHC1) SH2(PIK3R1) SH2(mix)
b
EGFR-NTEV-tcs-GV
Fluc (%)
Non-stimulated
Stimulated with 30 ng/mL EGF
FC: 1.18 1.87 1.79 1.94 2.02 1.89 1.28 1.89
ng/well: - 10 10 10 10 10 10 10
CTEV: Ctrl GRB2 SHC1 PIK3R1 SH2(GRB2) SH2(SHC1) SH2(PIK3R1) SH2(mix)
c
ERBB3-NTEV-tcs-GV | ERBB2
Fluc (%)
Non-stimulated
Stimulated with 10 ng/mL EGFld
FC: 0.39 2.53 2.37 2.87 2.61 1.57 1.84 1.52
ng/well: - 2.5 10 50 10 10 50 10
CTEV: Ctrl GRB2 SHC1 PIK3R1 SH2(GRB2) SH2(SHC1) SH2(PIK3R1) SH2(mix)
d
ERBB3-NTEV-tcs-GV | ERBB2
Fluc (%)
Non-stimulated
Stimulated with 10 ng/mL EGFld
FC: 0.95 2.06 2.06 2.32 3.91 1.67 2.94 1.45
ng/well: - 10 10 10 10 10 10 10
CTEV: Ctrl GRB2 SHC1 PIK3R1 SH2(GRB2) SH2(SHC1) SH2(PIK3R1) SH2(mix)
e
ERBB4-NTEV-tcs-GV
Fluc (%)
Non-stimulated
Stimulated with 10 ng/mL EGFld
FC: 0.99 1.94 1.81 1.77 1.96 1.61 1.15 0.94
ng/well: - 2.5 10 50 10 10 50 10
CTEV: Ctrl GRB2 SHC1 PIK3R1 SH2(GRB2) SH2(SHC1) SH2(PIK3R1) SH2(mix)
f
ERBB4-NTEV-tcs-GV
Fluc (%)
Non-stimulated
Stimulated with 10 ng/mL EGFld
FC: 1.27 2.14 2.33 2.27 2.56 1.36 1.57 1.64
ng/well: - 10 10 10 10 10 10 10
CTEV: Ctrl GRB2 SHC1 PIK3R1 SH2(GRB2) SH2(SHC1) SH2(PIK3R1) SH2(mix)
g
GRB2 SHC1 PI3KR1 SH2(GRB2) SH2(SHC1) SH2(PIK3R1) SH2(mix)
ng/well: 2.5 10 50 10 10 50 10
HA (ERBB3)
TUB
M (kDa) 100 70 50 40 35 50
h
CTEV-2HA: PI3KR1 SH2(PIK3R1) SHC1 SH2(SHC1) GRB2 SH2(GRB2) SH2(mix) blank
ng/well: 10 10 10 10 10 10 10
TUB
M (kDa) 100 70 50 40 35 50

Fig. S5. Transient RTK split TEV recruitment assays tolerate differences in transfected adapter plasmids.

a–f Split TEV recruitment assays for EGFR (**a**, **b**), ERBB2/ERBB3 (**c**, **d**), and ERBB4 (**e**, **f**). Note that for the ERBB2/ERBB3 assay (**c**), ERBB2 is co-transfected to allow heterodimerisation and thus ERBB3 phosphorylation, which is required for the recruitment of adapters. Assays were stimulated for 16 h, and analysed by a firefly luciferase assay. Non-stimulated samples are shown as open bars and stimulated once as grey bars. Ctrl, control (no adapter transfected). Non-stimulated samples are shown as open bars and stimulated once as grey bars. FC, fold change; Ctrl, control (no adapter transfected). Results are shown as average of 6 samples, error bars are shown as SEM. Significance was calculated using the unpaired t-test, with **, $p \leq 0.01$; ***, $p \leq 0.001$; ****, $p \leq 0.0001$; *n.s.* not significant.

g, **h** Biochemical validation of the expression of titrated (**g**) vs. non-titrated (**h**) adapters. Plasmids encoding adapter proteins (all tagged with CTEV-2HA) were transiently transfected into PC12 cells, allowed to express for 16 h, and lysed. Lysates were subjected to Western blotting using the indicated antibodies. Note that PIK3R1 is very low expressed in (**g**), whereas GRB2 is strongly expressed in (**h**). SH2(PIK3R1) is below detection limit in (**h**). Calculated sizes of fusion proteins are provided in Table S1.

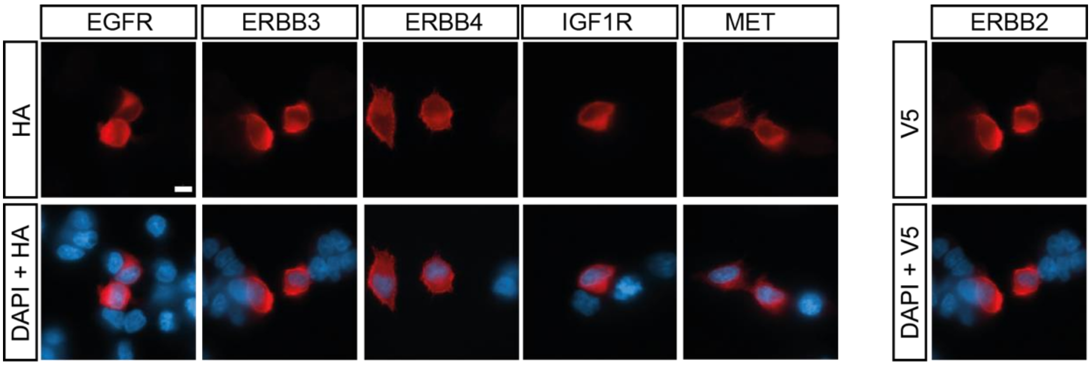

Fig. S6. RTK split TEV fusions are expressed at the cell membrane in PC12 cells.

RTK fusions are expressed at the cell membrane. Plasmids encoding EGFR, ERBB3, ERBB4, IGF1R, and MET (all tagged with NTEV-tcs-GV-2HA), ERBB2-V5, and adapter proteins (all tagged with CTEV-2HA) were transiently transfected into PC12 cells and fixed 20 h later for immunocytochemistry stainings. Receptor expression is displayed for EGFR-, ERBB3-, ERBB4-, IGF1R-, and MET-NTEV-tcs-GV-2HA (red, HA staining) as well as for ERBB2-V5 (red, V5 staining). Nuclei are displayed in blue (Dapi staining). Scale bar represents 5 µM.

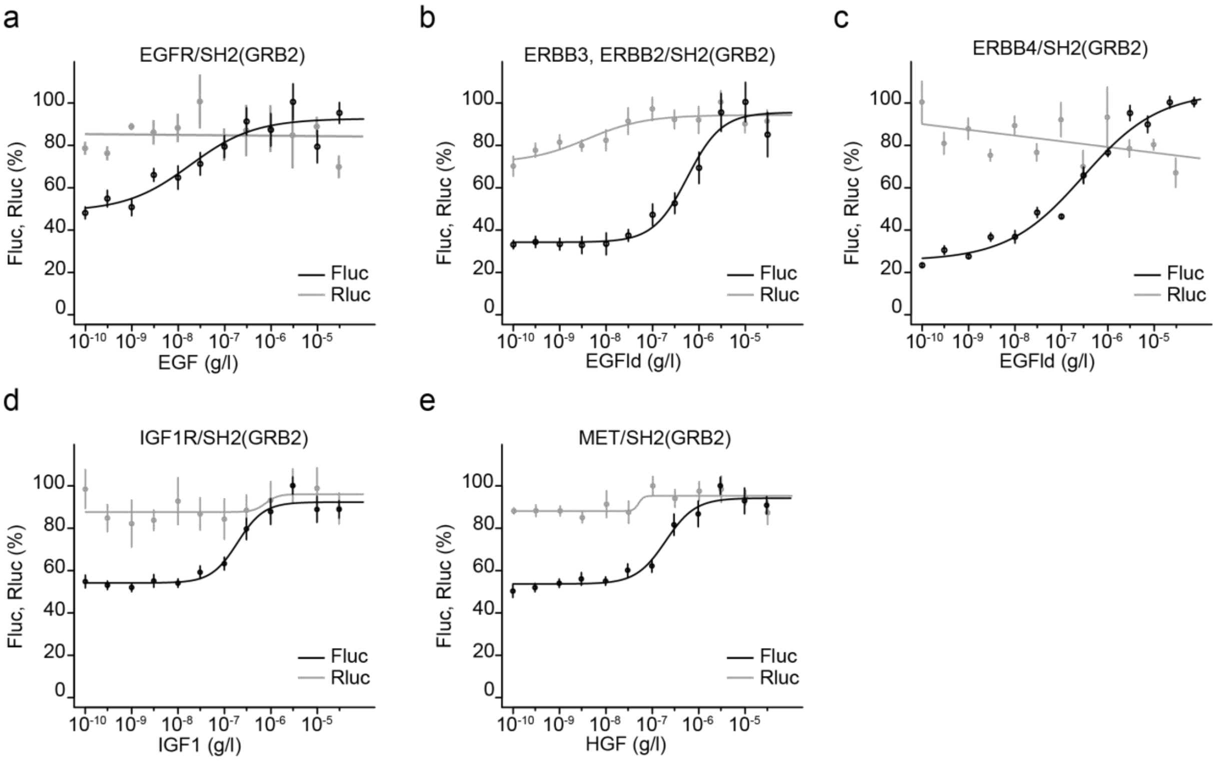

Fig. S7. Constitutive *Renilla* luciferase readout is stable at increasing ligand concentrations.

Split TEV recruitment assays monitoring the activity of EGFR (**a**), ERBB2/ERBB3 (**b**), ERBB4 (**c**), IGF1R (**d**), and MET (**e**). Each receptor fusion (NTEV-tcs-GV tag for EGFR, ERBB3, ERRB4, IGF1R, and MET; V5-tagged ERBB2) plasmid is co-transfected with the universal SH2(GRB2) CTEV adapter plasmid into PC12 cells. Depicted are dose–response curves, with agonists applied at increasing concentrations as indicated (EGF, EGFld, IGF1, HGF). Firefly luciferase is shown in black (as in Fig. 4), the constitutive *Renilla* luciferase signal is shown in grey. Error bars are shown as SEM, with 6 replicates per condition.

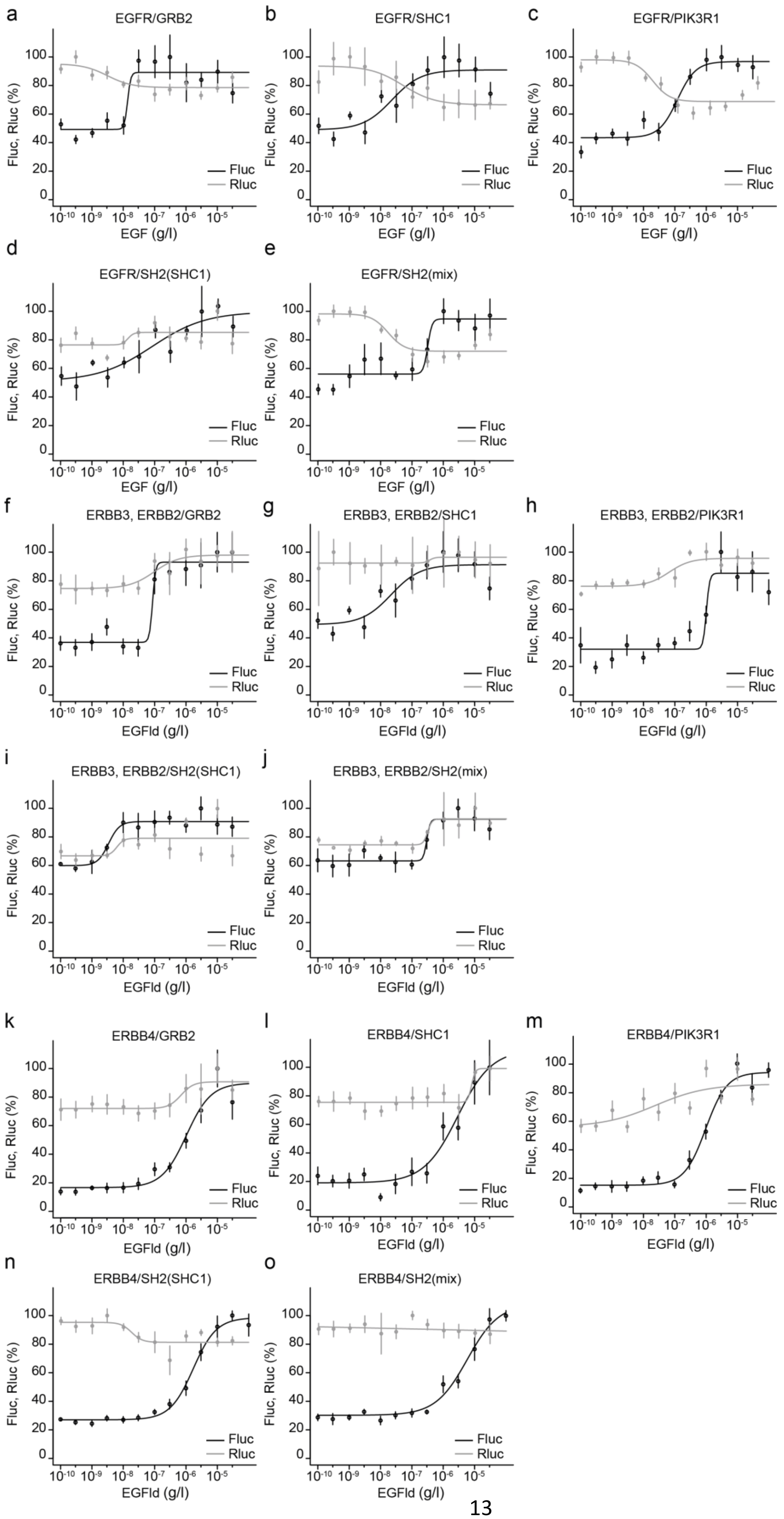
a
EGFR/GRB2
b
EGFR/SHC1
c
EGFR/PIK3R1
d
EGFR/SH2(SHC1)
e
EGFR/SH2(mix)
f
ERBB3, ERBB2/GRB2
g
ERBB3, ERBB2/SHC1
h
ERBB3, ERBB2/PIK3R1
i
ERBB3, ERBB2/SH2(SHC1)
j
ERBB3, ERBB2/SH2(mix)
k
ERBB4/GRB2
l
ERBB4/SHC1
m
ERBB4/PIK3R1
n
ERBB4/SH2(SHC1)
o
ERBB4/SH2(mix)
Fluc, Rluc (%)
EGF (g/l)
EGFld (g/l)
Fluc
Rluc

Fig. S8. Agonist dose–response data of split TEV recruitment assays for EGFR, ERBB2/ERBB3, and ERRB4 using full length and concatenated SH2 domain adapters.

EGFR (**a–e**), ERBB2/ERBB3 (**f–j**) and ERBB4 (**k–o**) split TEV recruitment assays were performed as dose–response assays in PC12 cells using full-length adapters of GRB2 (**a**, **f**, **k**), SHC1 (**b**, **g**, **l**), PIK3R1 (**c**, **h**, **m**), as well as SH2 domain adapters SH2(SHC1) (**d**, **i**, **n**) and SH2(mix) (**e**, **j**, **o**). Agonists EGF and EGF-like domain (EGFld) were applied at increasing concentrations as indicated. For each assay, plasmids encoding a receptor and an adapter, were co-transfected with a 10xUAS-minCMVp-driven firefly luciferase (Fluc) reporter plasmid shown in black and the constitutive *Renilla* luciferase is shown in grey, as follows:

(**a**) EGFR-NTEV-tcs-GV, GRB2-CTEV
(**b**) EGFR-NTEV-tcs-GV, SHC1-CTEV
(**c**) EGFR-NTEV-tcs-GV, PIK3R1-CTEV
(**d**) EGFR-NTEV-tcs-GV, SH2(SHC1)-CTEV
(**e**) EGFR-NTEV-tcs-GV, SH2(mix)-CTEV
(**f**) ERBB3-NTEV-tcs-GV, ERBB2, GRB2-CTEV
(**g**) ERBB3-NTEV-tcs-GV, ERBB2, SHC1-CTEV
(**h**) ERBB3-NTEV-tcs-GV, ERBB2, PIK3R1-CTEV
(**i**) ERBB3-NTEV-tcs-GV, ERBB2, SH2(SHC1)-CTEV
(**j**) ERBB3-NTEV-tcs-GV, ERBB2, SH2(mix)-CTEV
(**k**) ERBB4-NTEV-tcs-GV, GRB2-CTEV
(**l**) ERBB4-NTEV-tcs-GV, SHC1-CTEV
(**m**) ERBB4-NTEV-tcs-GV, PIK3R1-CTEV
(**n**) ERBB4-NTEV-tcs-GV, SH2(SHC1)-CTEV
(**o**) ERBB4-NTEV-tcs-GV, SH2(mix)-CTEV

Error bars represent SEM, with 6 replicates per condition.

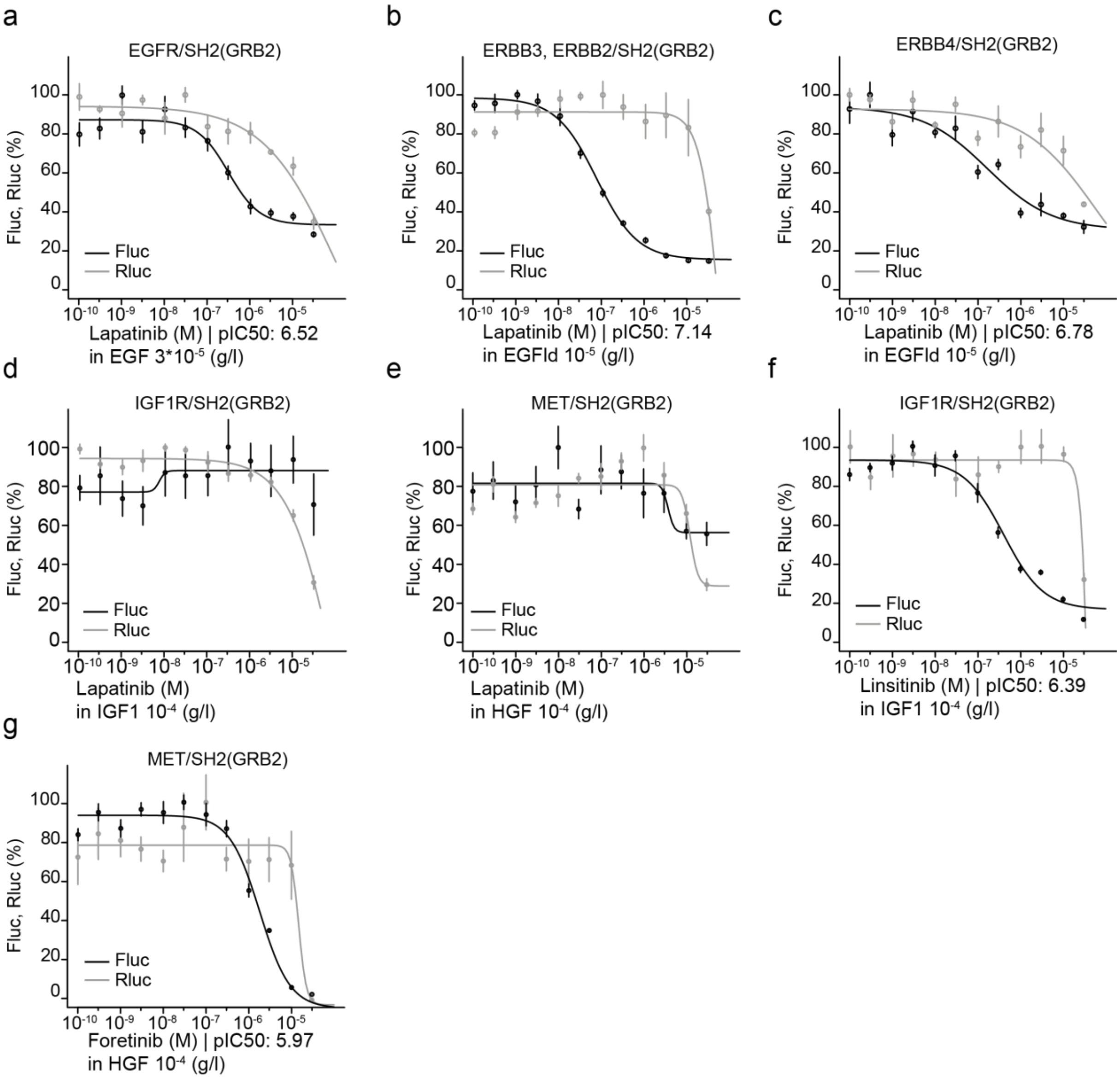

Fig. S9. Antagonists lapatinib, linsitinib and foretinib inhibit their cognate RTK targets.

a–d Split TEV recruitment assays monitoring the lapatinib-mediated inhibition of EGFR (**a**), ERBB2/ERBB3 (**b**), and ERBB4 (**c**), but not IGFR1 (**d**) nor MET (**e**). **f**, **g** Linsitinib-mediated inhibition of IGF1R (**d**), and foretinib-mediated inhibition of MET (**e**). Each receptor fusion (NTEV-tcs-GV tag for EGFR, ERBB3, ERRB4, IGF1R, and MET; V5-tagged ERBB2) plasmid is co-transfected with the universal SH2(GRB2)-CTEV adapter plasmid into PC12 cells as indicated. Depicted are dose–response curves with a constant agonist (EGF, EGFld, IGF1, HGF) and increasing concentrations of lapatinib (**a–d**), linsitinib (**e**) and foretinib (**g**). Firefly luciferase is shown in black (as in Fig. 5), the constitutive *Renilla* luciferase is shown in grey. Note that the constitutive *Renilla* luciferase readout indicates toxicity of lapatinib, linsitinib, and foretinib at 30 µM. Error bars are shown as SEM, with 6 replicates per condition.

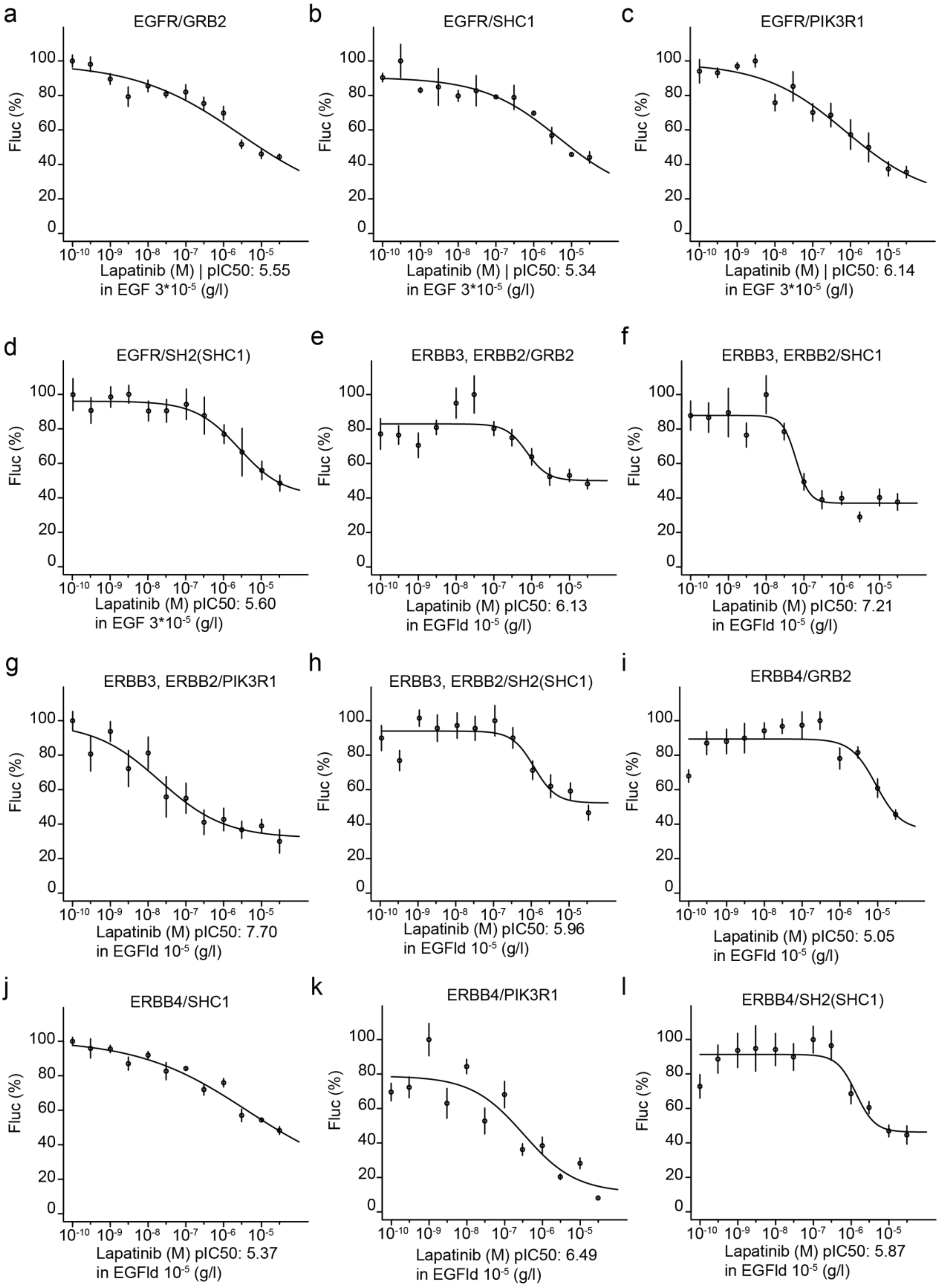

Fig. S10. Dose–response data of ERBB family split TEV recruitment assays using full length and concatenated SH2 domain adapters testing lapatinib inhibition.

EGFR (**a–d**), ERBB2/ERBB3 (**e–h**) and ERBB4 (**i–l**) split TEV recruitment assays were performed as dose–response assays in PC12 cells using full-length adapters of GRB2 (**a**, **e**, **i**), SHC1 (**b**, **f**, **j**), PIK3R1 (**c**, **g**, **k**), and the SH2 domain adapter SH2(SHC1) (**d**, **h**, **l**). The ERBB family antagonist lapatinib was applied at increasing concentrations as indicated. Agonists EGF and EGF-like domain (EGFld) were applied at constant concentrations as indicated. For each assay, plasmids encoding a receptor and an adapter, were co-transfected with a 10xUAS-minCMVp-driven firefly luciferase (Fluc) reporter plasmid, as follows:

(**a**) EGFR-NTEV-tcs-GV, GRB2-CTEV
(**b**) EGFR-NTEV-tcs-GV, SHC1-CTEV
(**c**) EGFR-NTEV-tcs-GV, PIK3R1-CTEV
(**d**) EGFR-NTEV-tcs-GV, SH2(SHC1)-CTEV
(**e**) ERBB3-NTEV-tcs-GV, ERBB2, GRB2-CTEV
(**f**) ERBB3-NTEV-tcs-GV, ERBB2, SHC1-CTEV
(**g**) ERBB3-NTEV-tcs-GV, ERBB2, PIK3R1-CTEV
(**h**) ERBB3-NTEV-tcs-GV, ERBB2, SH2(SHC1)-CTEV
(**i**) ERBB4-NTEV-tcs-GV, GRB2-CTEV
(**j**) ERBB4-NTEV-tcs-GV, SHC1-CTEV
(**k**) ERBB4-NTEV-tcs-GV, PIK3R1-CTEV
(**l**) ERBB4-NTEV-tcs-GV, SH2(SHC1)-CTEV

Error bars represent SEM, with 6 replicates per condition.

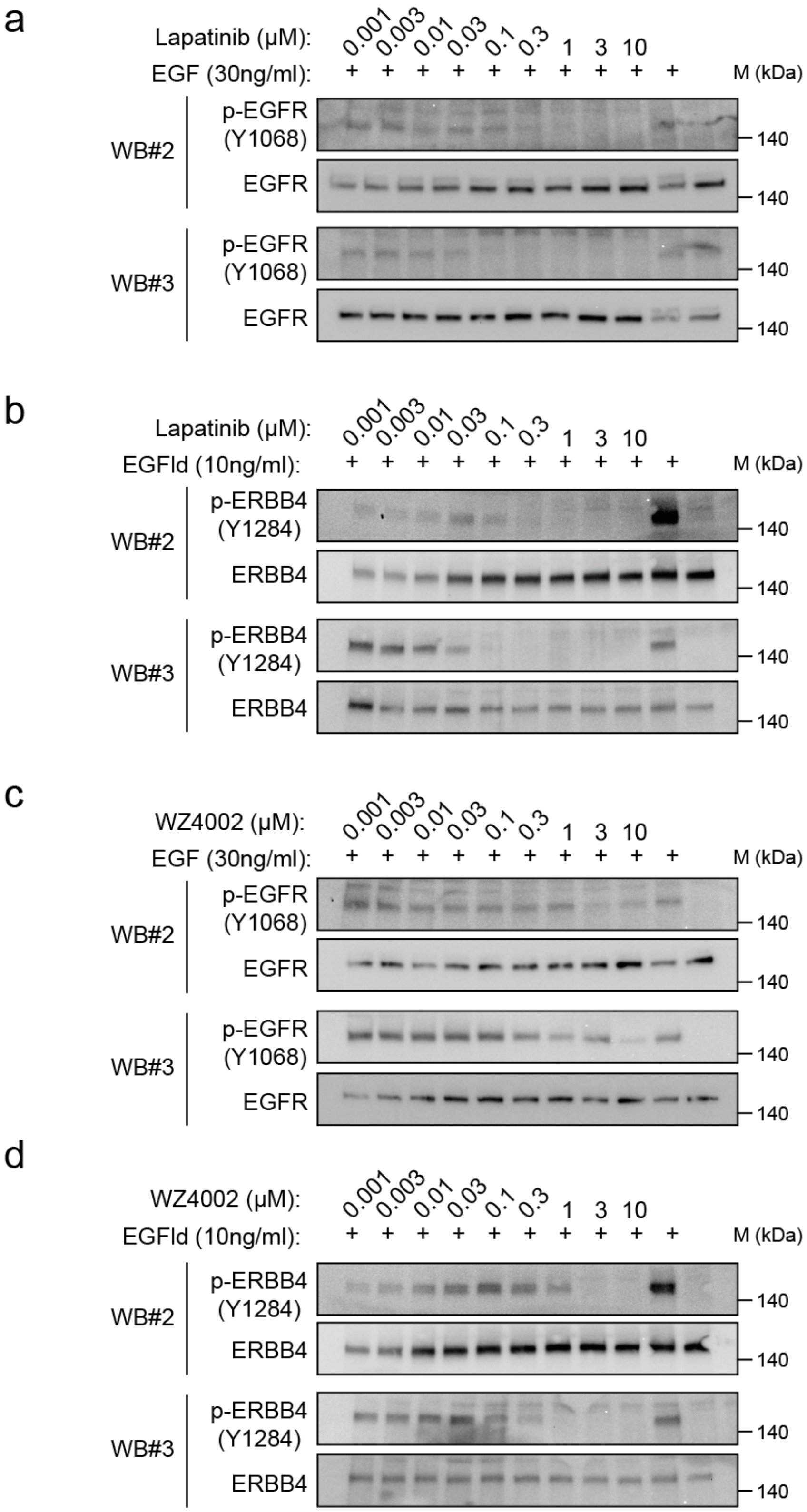

Fig. S11. Lapatinib and WZ4002 reduce p-EGFR (Y1068) and p-ERBB4 (Y1284) levels in A549 cells and T-47D cells.

Starved cells were treated for 1 h with increasing concentrations of lapatinib (**a**, **b**) or WZ4002 (**c**, **d**) and stimulated for 5 mins with 30 ng/ml EGF (**a**, **c**) or 10 ng/ml EGFld (**b**, **d**) where indicated. Lysates were subjected to Western blotting and probed with indicated antibodies.

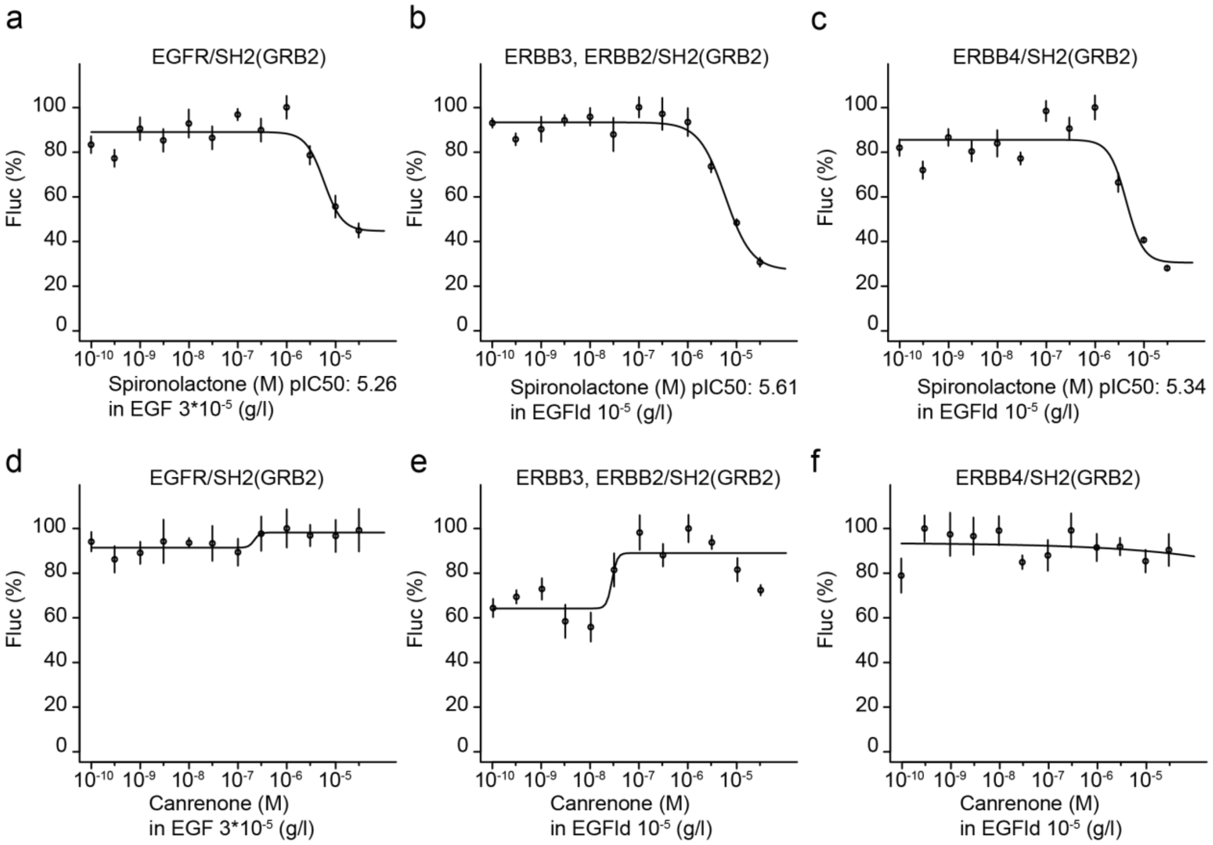

Fig. S12. Spironolactone inhibits ERBB receptor activities using the universal SH2 domain adapter in the split TEV recruitment assay.

Antagonist dose–response split TEV recruitment assays using spironolactone (**a–c**) and canrenone (**d–f**), which were both applied at increasing concentrations as indicated. EGFR (**a**, **d**), ERBB2/ERBB3 (**b**, **e**), and ERBB4 (**c**, **f**) split TEV recruitment assays were performed in PC12 cells using the SH2 domain adapter SH2(GRB2). Agonists EGF and EGF-like domain (EGFld) were applied at constant concentrations as indicated. For each assay, plasmids encoding a receptor and an adapter, were co-transfected with a 10xUAS-minCMVp-driven firefly luciferase (Fluc) reporter plasmid, as follows:

(**a**) EGFR-NTEV-tcs-GV, SH2(GRB2)-CTEV
(**b**) ERBB3-NTEV-tcs-GV, ERBB2, SH2(GRB2)-CTEV
(**c**) ERBB4-NTEV-tcs-GV, SH2(GRB2)-CTEV
(**d**) EGFR-NTEV-tcs-GV, SH2(GRB2)-CTEV
(**e**) ERBB3-NTEV-tcs-GV, ERBB2, SH2(GRB2)-CTEV
(**f**) ERBB4-NTEV-tcs-GV, SH2(GRB2)-CTEV

Error bars represent SEM, with 6 replicates per condition.

Supplementary Tables

Table S1. Calculation of p-values using an unpaired t-test for assay data shown in Fig. 2.

Receptor-NTEV-tcs-GV<br>Adapter-CTEV	EGFR	ERBB2/<br>ERBB3	ERBB4	IGF1R	MET
Control	0.4756	0.1443	0.9645	0.790	0.3153
GRB2	0.0137	<0.0001	<0.0001	0.0016	0.0002
SHC1	0.0005	0.0003	0.0006	0.0004	0.0406
PIK3R1	0.0032	<0.0001	0.0064	<0.0001	0.0017
SH2(GRB2)	<0.0001	<0.0001	<0.0001	<0.0001	0.0004
SH2(SHC1)	0.0390	0.0048	0.0182	0.0716	0.0009
SH2(PIK3R1)	0.1228	0.0207	0.1822	0.0042	0.0897
SH2(mix)	0.0079	0.0088	0.7840	0.0224	0.8422

The unpaired t-test was run using GraphPad Prism (version 5).

Table S2. Calculated sizes of fusion proteins.

Fusion protein	Size (kDa)
EGFR-NTEV-tcs-GV-2HA	187.4
ERBB2-V5	141.2
ERRB3-NTEV-tcs-GV-2HA	201.3
ERBB4-NTEV-tcs-GV-2HA	200.1
IGF1R-NTEV-tcs-GV-2HA	208
MET-NTEV-tcs-GV-2HA	210.9
SHC1-CTEV-2HA	67.4
PIK3R1-CTEV-2HA	99.3
GRB2-CTEV-2HA	40.9
SH2(GRB2)-CTEV-2HA	49.9
SH2(SHC1)-CTEV-2HA	48.4
SH2(PIK3R1)-CTEV-2HA	50.3
SH2(mix)-CTEV-2HA	49.5

Receptors were fused to NTEV-tcs-GV-2HA, adapters to CTEV-2HA.

kDa kilodaltons

Table S3. IC_{50} and pIC_{50} values for lapatinib obtained from dose–response split TEV recruitment assays as indicated.

**Receptor-NTEV-tcs-GV Adapter-CTEV**	**EGFR**		**ERBB2/ERBB3**		**ERBB4**	
	**IC_{50} [μM]**	**pIC_{50}**	**IC_{50} [μM]**	**pIC_{50}**	**IC_{50} [μM]**	**pIC_{50}**
GRB2	2.791	5.55	0.747	6.13	9,005	5.05
SHC1	4.561	5.34	0.062	7.21	4.275	5.37
PIK3R1	0.730	6.14	0.020	7.70	0.321	6.49
SH2(GRB2)	0.305	6.52	0.072	7.14	0.166	6.78
SH2(SHC1)	2.511	5.60	1.091	5.96	1.356	5.87
